# A triple distinction of cerebellar function for oculomotor learning and fatigue compensation

**DOI:** 10.1371/journal.pcbi.1011322

**Published:** 2023-08-04

**Authors:** Jana Masselink, Alexis Cheviet, Caroline Froment-Tilikete, Denis Pélisson, Markus Lappe

**Affiliations:** 1 Institute for Psychology & Otto Creutzfeldt Center for Cognitive and Behavioral Neuroscience, University of Münster, Münster, Germany; 2 IMPACT Team, Lyon Neuroscience Research Center, University Claude Bernard Lyon 1, Bron cedex, France; 3 Department of Psychology, Durham University, South Road, Durham, United Kingdom; 4 Hospices Civils de Lyon—Pierre-Wertheimer Hospital, Neuro-Ophtalmology Unit, Bron cedex, France; Queen’s University, CANADA

## Abstract

The cerebellum implements error-based motor learning via synaptic gain adaptation of an inverse model, i.e. the mapping of a spatial movement goal onto a motor command. Recently, we modeled the motor and perceptual changes during learning of saccadic eye movements, showing that learning is actually a threefold process. Besides motor recalibration of (1) the inverse model, learning also comprises perceptual recalibration of (2) the visuospatial target map and (3) of a forward dynamics model that estimates the saccade size from corollary discharge. Yet, the site of perceptual recalibration remains unclear. Here we dissociate cerebellar contributions to the three stages of learning by modeling the learning data of eight cerebellar patients and eight healthy controls. Results showed that cerebellar pathology restrains short-term recalibration of the inverse model while the forward dynamics model is well informed about the reduced saccade change. Adaptation of the visuospatial target map trended in learning direction only in control subjects, yet without reaching significance. Moreover, some patients showed a tendency for uncompensated oculomotor fatigue caused by insufficient upregulation of saccade duration. According to our model, this could induce long-term perceptual compensation, consistent with the overestimation of target eccentricity found in the patients’ baseline data. We conclude that the cerebellum mediates short-term adaptation of the inverse model, especially by control of saccade duration, while the forward dynamics model was not affected by cerebellar pathology.

## Author summary

Achieving a fine-grained understanding of how the cerebellum continuously recalibrates our movements is an ongoing challenge in sensorimotor neuroscience. Recently, we showed that recalibration of saccadic eye movements does not only operate in motor space, i.e. by adjusting the motor command, but also in external and internal visual space, i.e. by adjusting the spatial representation of the target and the internal saccade size. For this purpose, (1) we developed a paradigm that allowed us to monitor changes of the internal saccade size estimated from trans-saccadic target localizations, and (2) we unified the three learning processes in one computational modeling framework. Here we apply this approach to the saccade learning data of patients with a neurodegenerative cerebellar disease. First, we dissociate the cerebellar role in recalibration of the three sites of learning. Second, we show how learning is transposed to saccade kinematics. Third, we provide first insights into the perceptual consequences of cerebellar pathology that, according to our model, may be a mechanism to recover from disease-specific motor deficits. Our modeling framework may help to dissociate the contribution of specific sensorimotor areas to adaptive behavior as well as to improve the understanding of learning deficits and compensatory strategies in the clinical context.

## Introduction

The cerebellum modulates supervised error-based learning [1–3]. If a movement is inaccurate, an error signal adapts the synaptic gains of cerebellar Purkinje cells [4–7] that implement an inverse model, i.e. the mapping of the visuospatial movement goal onto a motor command [4, 8, 9]. If e.g. the eye muscles fatigue, the synaptic gains are modulated such that the saccade does not fall short of the target [10–12]. To compensate for continuous fluctuations of muscle dynamics, the cerebellum is constantly recalibrating the saccadic circuitry [13–15].

However, the traditional view on the cerebellum as a pure motor hub has changed. The cerebellum is interconnected with almost the entire cortex [16–18], including retinotopic areas [19–21], and basal ganglia [18], and holds at least five visuospatial maps itself [22]. Moreover, it has been shown that saccadic motor learning also comprises changes in visuospatial representations. These changes occur, firstly, in the visuospatial target map when errors are assigned to an internal failure of spatial target representation [23–27]. Secondly, these changes occur in the internal representation of saccade size as measured by trans-saccadic target localizations [25, 28, 29].

Changes in the internal saccade representation affect the forward dynamics model that transforms the corollary discharge, i.e. a copy of the motor command (*CD*_*M*_) [30–32] into a visuospatial representation of saccade size (*CD*_*V*_) [4, 33, 34]. Intact *CD*_*V*_ information is necessary to correctly bridge the spatial gap between pre- and post-saccadic visual input that supports our perception of a stable world [35–37]. In Masselink et al. (2021) [38], we implemented the action-perception entanglement during learning in a computational model that collectively adapts the visuospatial target map, the inverse model and the forward dynamics model in response to postdictive motor error, i.e. motor error with respect to a postdicted target position.

Cerebellar perturbations restrain saccade changes during learning, as shown e.g. in lesioned monkeys [39, 40], in cerebellar patients [41–43] and in healthy subjects with non-invasive cerebellar stimulation [44–46]. Because of its circular learning architecture, its visuospatial maps and its CD projection pathway to frontal cortex [47], the cerebellum is an ideal candidate to also recalibrate the visuospatial target map and the forward dynamics model.

In Cheviet et al. (2022) [48] we found first empirical evidence that changes in visual target localizations during saccadic motor learning are impaired in cerebellar patients. These target localizations were performed (1) during fixation and (2) trans-saccadically after a saccade landed in the dark. The dataset includes eight patients with a neurodegenerative cerebellar disease and eight healthy control subjects who performed three conditions comprising (1) learning from peri-saccadic inward and (2) from peri-saccadic outward target steps, and (3) compensation of neural oculomotor fatigue, i.e. without target step. As learning relies on plasticity of the transformation processes between visual and motor representations (i.e. on how the target is scaled on the visuospatial map, on the inverse model and on the forward dynamics model), a quantification of the relative impairments of these transformations is essential to draw conclusions on cerebellar function.

Here we apply an expanded version of the visuomotor learning model of Masselink et al. (2021) [38] to the empirical data of Cheviet et al. (2022) [48] in order to quantify the recalibration of the visuospatial target map, of the inverse model and of the forward dynamics model in cerebellar patients. To achieve a deeper understanding of which control processes are perturbed, we expanded the model of Masselink et al. (2021) [38] with (1) how changes in the motor command are transposed to saccade kinematics (i.e. saccade peak velocity and saccade duration), and (2) how the visuomotor system compensates for a fatigue-induced decline in saccade peak velocity. Our results reveal that cerebellar pathology reduces short-term learning of the inverse model, especially when controlled by upregulation of saccade duration. Notably, the forward dynamics model and thus, the *CD*_*V*_ signal, seemed correctly informed about the reduced saccade change. Learning of the visuospatial target map trended in learning direction only in control subjects, yet without significance. Moreover, we show that cerebellar pathology is accompanied by an overestimation of target eccentricity that, according to our model, could stem from error reduction to counteract oculomotor fatigue on a long timescale. We conclude that intact recalibration of the inverse model, particularly via control of saccade duration, depend on cerebellar integrity. The forward dynamics model, at least for learning from post-saccadic errors, is not affected by cerebellar pathology. Long-term learning at perceptual level may partially compensate for cerebellar motor deficits.

## Results

To reveal how adaptation of the visuospatial target map, of the inverse model and of the forward dynamics model for saccade motor learning relies on cerebellar integrity, we fitted a visuomotor learning model to the saccade and visual target localization data of Cheviet et al. (2022) [48], including eight cerebellar patients and eight healthy control subjects. Table 1 lists the characteristics of the cerebellar patients, including disease type, disease duration and anatomical involvements.

**Table 1:** Disease types and anatomical involvements of cerebellar patients.

Patient	Age	Sex	Disease	Anatomical involvement	Disease duration (years)
P1	57	m	Spinocerebellar ataxia, type 1	Hemispheric cerebellar atrophy	21
P2	59	f	Friedreich ataxia	Discrete superior & posterior vermal atrophy	7
P3	48	f	Spinocerebellar ataxia, type 3	Hemispheric cerebellar atrophy	13
P4	44	m	Unknown autosomic dominant inherited cereb. ataxia	Vermal & superior cereb. atrophy	15
P5	43	m	Spinocerebellar ataxia, type 28	Discrete global cerebellar atrophy	9
P6	70	m	Fragile X–associated tremor/ataxia syndrome	Discrete vermal & hemispheric cereb. atrophy	8
P7	63	f	Late onset sporadic ataxia	Discrete vermal atrophy	2
P8	60	f	Friedreich ataxia	Superior & posterior vermal atrophy	20

### Experimental data

**Fig 1:**
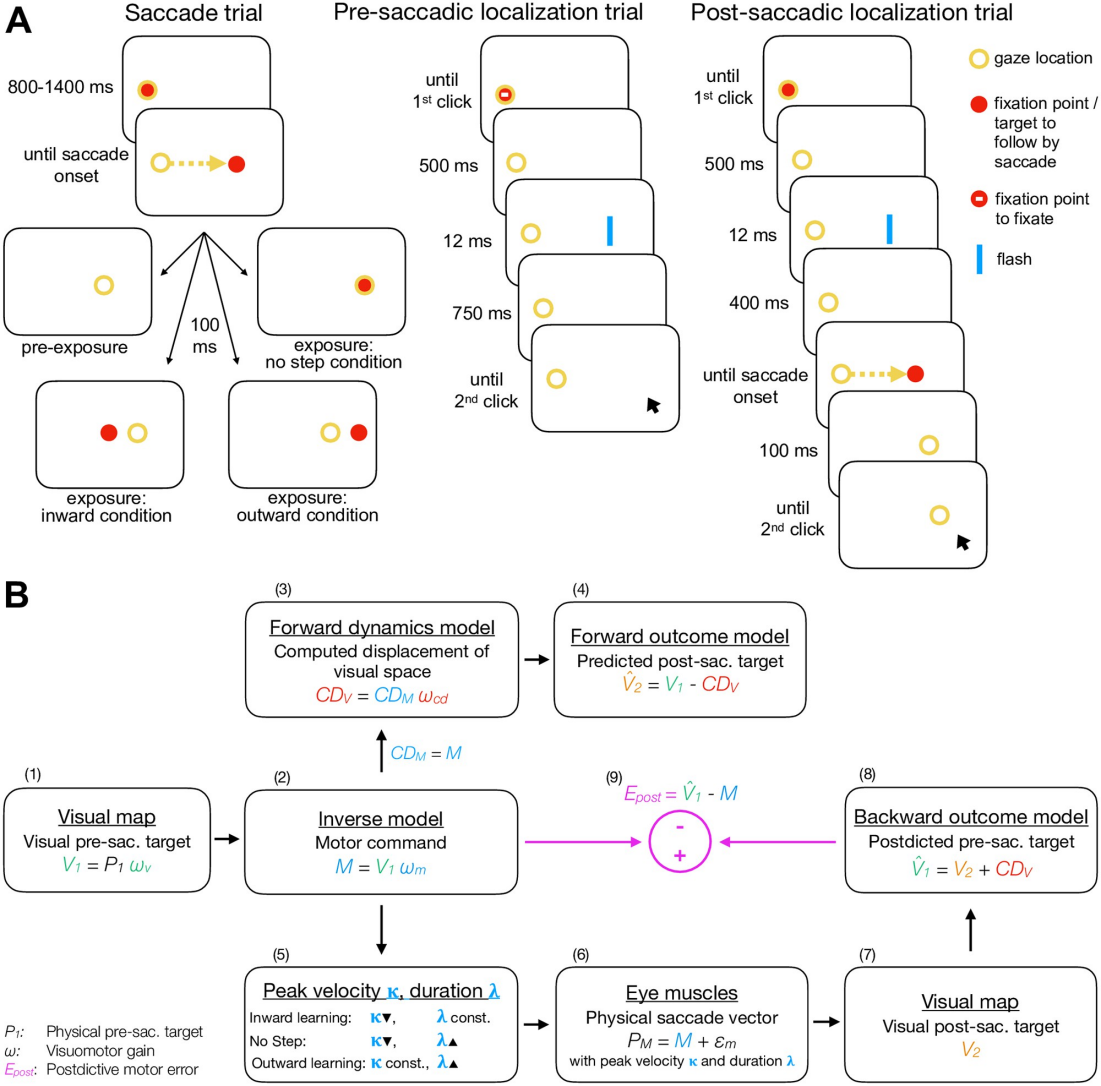
Experimental tasks and model framework. **(A)** In the saccade trials, subjects executed a saccade to a 20° rightward target. In the pre-exposure phase, the target was extinguished at saccade onset. In the exposure and post-exposure phase, the target either stepped 6° inward (inward condition), 6° outward (outward condition) or stayed at its initial position (no step condition). In the pre-saccadic localization trials, subjects localized a 12 ms flash with a mouse cursor while holding gaze at the fixation point. In the post-saccadic localization trials, subjects performed a saccade to a 20° rightward target and then localized the 12 ms pre-saccadic flash with a mouse cursor. Please note that the stimuli are not drawn to scale. The mouse cursor was a blue line pointer. The yellow circle illustrates gaze location but was not present at the stimulus display. **(B)** The target with the physical distance *P*_1_ is represented at the location *V*_1_ on the visuospatial map. An inverse model maps *V*_1_ onto a motor command *M*. Before saccade start, a forward dynamics model transforms a copy of the motor command *CD*_*M*_ into visuospatial coordinates, i.e. into the computed displacement of visual space *CD*_*V*_. A forward outcome model then shifts retinal coordinates by *CD*_*V*_ to predict the visual location of the post-saccadic target ${\hat{V}}_{2}$. For saccade execution, the motor command is transposed to saccade peak velocity *κ* and saccade duration λ, producing the saccade vector *P*_*M*_. The actual post-saccadic target appears at retinal position *V*_2_. A backward outcome model then postdicts the visual post-saccadic target back to pre-saccadic space ${\hat{V}}_{1}$. The visuomotor system evaluates the accuracy of the motor command with respect to the postdicted target position (*E*_*post*_) in order to adapt its gains *ω*_*v*_, *ω*_*m*_ and *ω*_*cd*_. A decrease of *ω*_*m*_ (inward learning) is controlled by downregulating saccade peak velocity while an increase of *ω*_*m*_ (outward learning) is controlled by upregulation of saccade duration. When saccades are performed repetitively to the same, non-stepping target, oculomotor fatigue occurs, i.e. peak velocity declines, which is usually compensated by an increase of saccade duration.

Each subject participated in three conditions requiring saccades to a 20° rightward target as well as pre- and post-saccadic visual localizations of a shortly flashed bar presented around the usual target position (Fig 1A). In the pre-saccadic localization trials, subjects localized the flash with a mouse cursor while holding gaze at the fixation point. In the post-saccadic localization trials, subjects performed a saccade to a 20° rightward target and then localized the pre-saccadic flash that had appeared before target onset. The pre- and the post-exposure phase (phase 1 and 3) measured subjects’ state of saccade vector, pre- and post-saccadic visual localizations before and after exposure. The exposure phase (phase 2) induced either (1) saccade shortening in response to a 6° peri-saccadic inward target step (inward condition), (2) saccade lengthening in response to a 6° peri-saccadic outward target step (outward condition), or (3) tested the ability to maintain the saccade vector during repetitive, stereotyped saccades to a non-stepping target (no step condition). This task is known to induce oculomotor fatigue, i.e. a decline in saccade peak velocity. In healthy subjects, this is usually counteracted by upregulation of saccade duration such that the saccade vector stays stable [49–55]. Compared to learning in response to an assumed fatigue of the eyes muscles, oculomotor fatigue is of neural origin and perhaps due to a loss of motivation or attention [55]. However, patients with cerebellar lesion of the vermis have shown to be impaired in compensating oculomotor fatigue [42].

**Fig 2:**
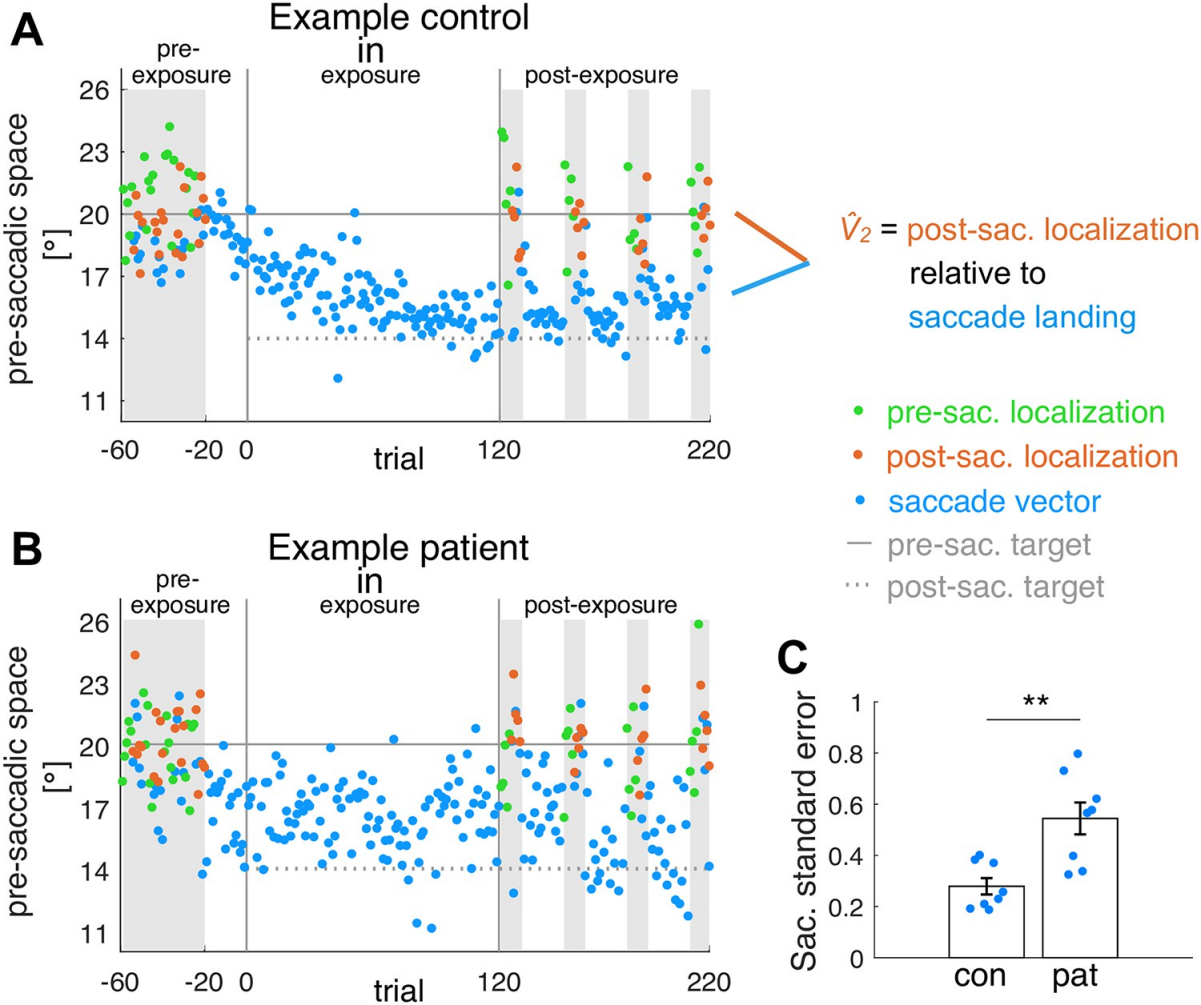
Example subject data for the inward condition and within-subject saccade variability in the pre-exposure phase. Saccade vectors, pre- and post-saccadic localizations for the inward condition of **(A)** an example control subject and **(B)** an example patient. The pre-exposure phase measured subjects’ baseline state without target step. The exposure phase induced saccadic learning and the post-exposure phase measured adaptation-induced changes to the saccade vector, pre- and post-saccadic localizations. Similar to the pattern that we found at group level, the patient shows less learning in the saccade vector and higher saccade endpoint variability compared to the control subject. In the model, the predicted post-saccadic target ${\hat{V}}_{2}$ is derived from post-saccadic localization relative to saccade landing. **(C)** Within-subject standard error of saccade vectors in the pre-exposure phase (averaged across the three conditions per subject) compared between control subjects and patients, *** *p* <.001, ** *p* <.01, * *p* <.05 and n.s. *p* ≥.05.

**Fig 3:**
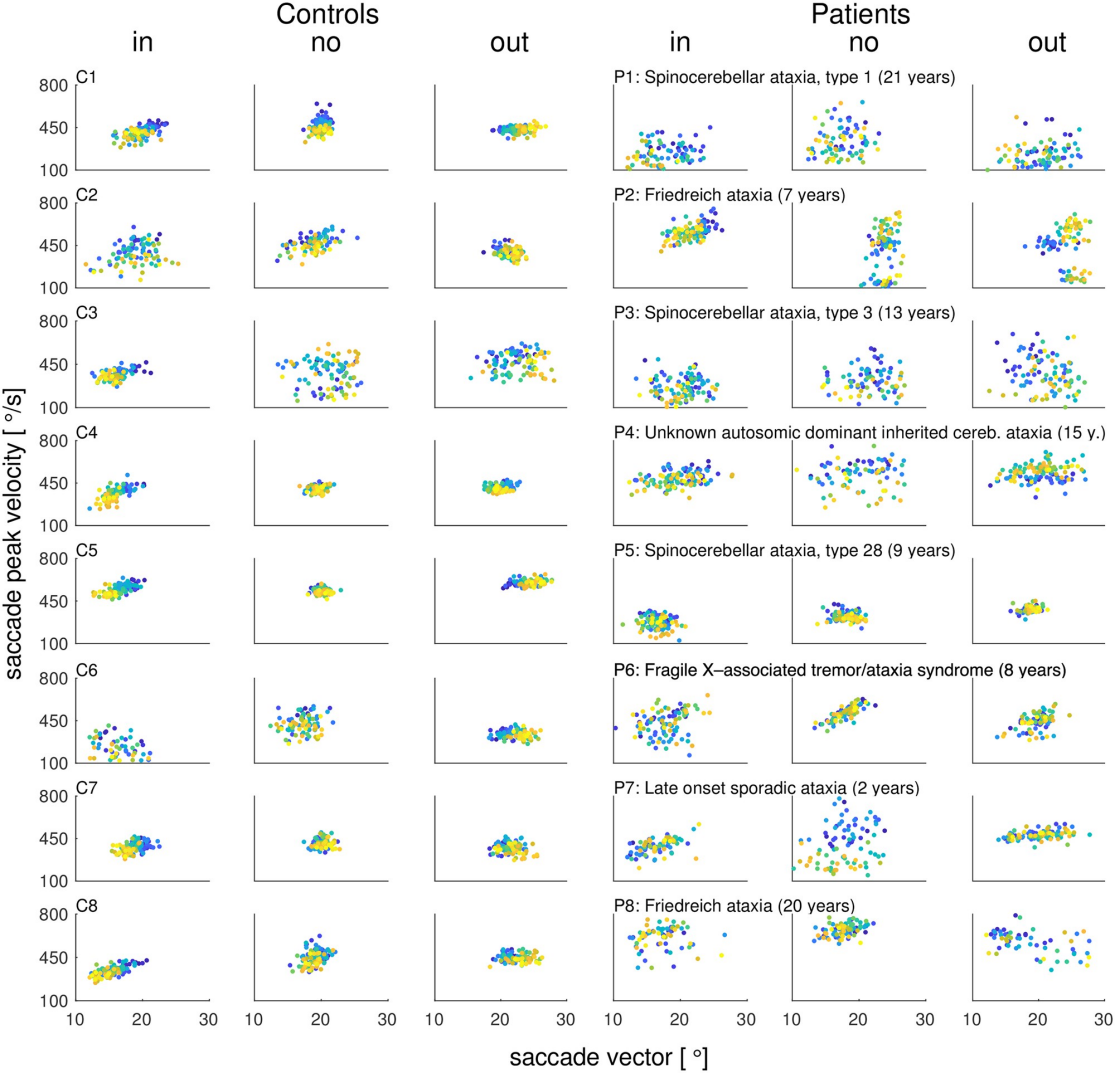
Individual saccade peak velocities in dependence on saccade vectors from early trials (blue) to late trials (yellow). Saccade peak velocities and saccade vectors are shown separately for each subject and condition (controls C1-C8, patients P1-P8 including disease type and disease duration in years). During inward learning of control subjects, the decrease of the saccade vector encompasses a decrease of saccade peak velocity. This is in line with saccade shortening being mainly controlled by downregulating peak velocity. During outward learning, the saccade vector increases while peak velocity stays stable. This is in line with saccade lengthening being mainly controlled by upregulating saccade duration (see Fig 4). In the no step condition, saccade peak velocity encompasses a small but substantial decline that, however, is not accompanied by a decline of the saccade vector. This is a sign of oculomotor fatigue being successfully compensated by healthy subjects. As known from cerebellar patients, the patients’ saccade vectors and peak velocities are more variable than in control subjects. Moreover, overall learning effects on saccade vector and peak velocity are smaller. In the no step condition, patients show substantial oculomotor fatigue, i.e. a decline in peak velocity that is not fully compensated by saccade duration.

**Fig 4:**
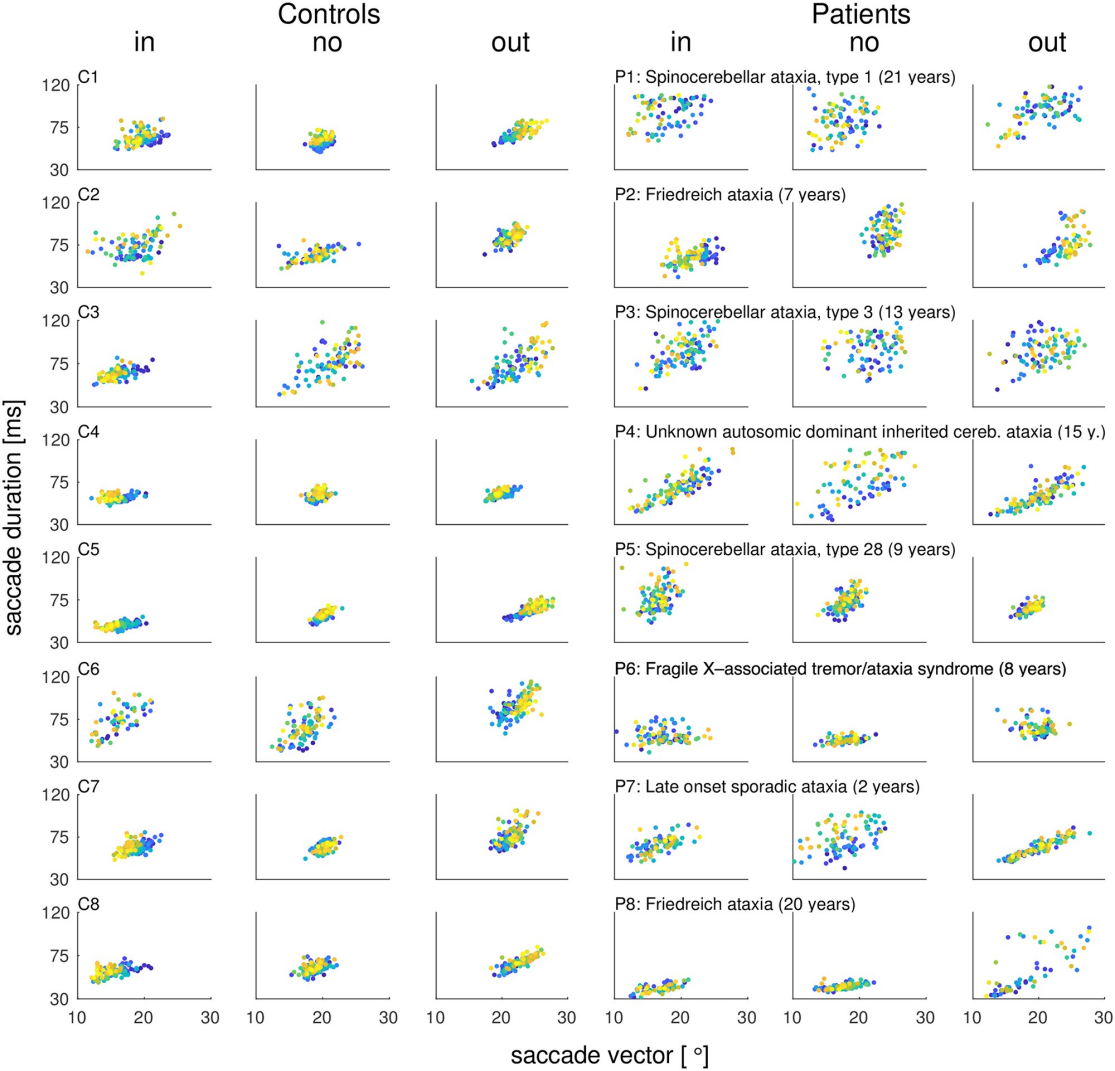
Individual saccade durations in dependence on saccade vectors from early trials (blue) to late trials (yellow). Saccade durations and saccade vectors are shown separately for each subject (controls C1-C8, patients P1-P8) and condition. During inward learning of control subjects, saccade duration declines in subjects C3, C5, C7 and C8 (yet not significantly across subjects when compared between the pre- and the post-expose phase, *t*_7_ = -1.01, *p* = .346). The decrease of the saccade vector rather stems from a decline of saccade peak velocity (see Fig 3). In the outward condition, saccade duration increases together with the saccade vector. This is consistent with saccade lengthening being mainly controlled by upregulation of saccade duration. In the no step condition, saccade duration increases, thereby compensating for the peak velocity loss seen in Fig 3. In patients, small learning effects can be seen but saccade vectors and durations are more noisy. In the no step condition, saccade duration is increased only in patient P4 and P7. Overall, duration cannot sufficiently counteract the peak velocity loss.

Fig 2A shows the inward learning data of an example control subject and Fig 2B shows the inward learning data of an example patient. Amplitude adaptation is impaired in the patient compared to the control subject. Fig 2C shows that saccade variability was higher in patients than in control subjects. An increased variability of saccade endpoints has been reported before as a side-effect of cerebellar dysfunction [40, 42, 43, 56]. The standard error of saccade vectors in the pre-exposure phase averaged across the three conditions was 0.28 ± 0.03° in control subjects and 0.54 ± 0.06° in patients (difference between patients and control subjects *t*_14_ = -3.78, *p* = .002). Moreover, the mean saccade vector in the pre-exposure phase averaged across the three conditions differed more between patients than between control subjects (cross-subject standard error of saccade vectors, control subjects 0.37°, patients 0.57°). Fig 3 shows the individual saccade peak velocities and Fig 4 the individual saccade durations in dependence on saccade vector for control subjects (C1-C8) and patients (P1-P8) from early trials (blue) to late trials (yellow).

Please note that because the analysis of the empirical data has been presented in Cheviet et al. (2022) [48], this paper focuses on a model-based analysis.

### Model fits

**Fig 5:**
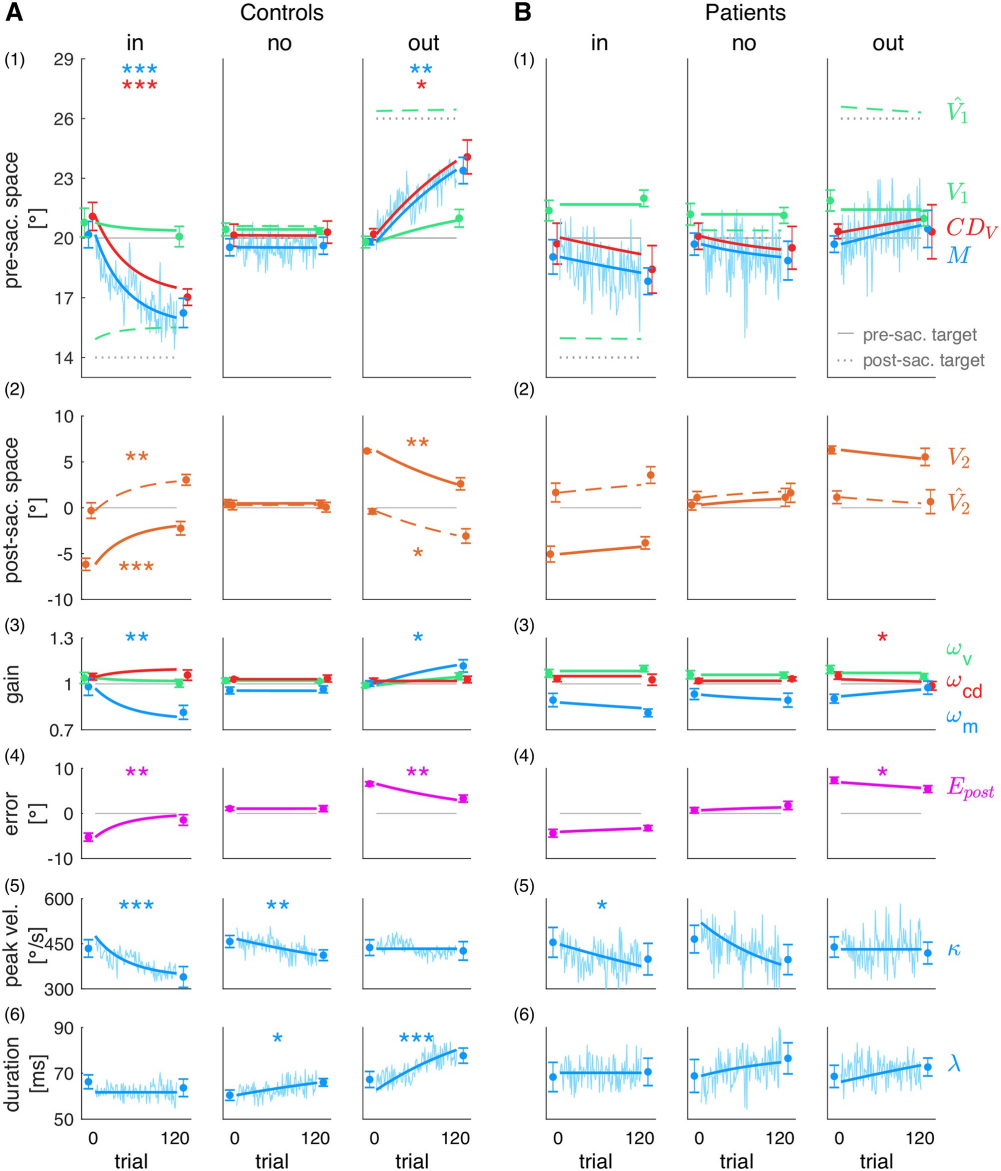
Model fits to the group data. Pre- and post-exposure mean ± standard error (dots with error bars), trial-by-trial saccade data of the exposure phase (jagged blue lines) and model fits to the data (smooth lines) for **(A)** control subjects and **(B)** cerebellar patients, separately for each condition (inward, no step, outward). (1) Visual pre-saccadic target *V*_1_ (fitted to pre-saccadic localizations), motor command *M* (fitted to saccade vectors), computed displacement of visual space *CD*_*V*_, postdicted pre-saccadic target ${\hat{V}}_{1}$. (2) Predicted post-saccadic target ${\hat{V}}_{2}$ (fitted to post-saccadic localizations with respect to saccade landing), visual post-saccadic target *V*_2_. (3) Visual gain *ω*_*v*_, motor gain *ω*_*m*_, CD gain *ω*_*cd*_. (4) Postdictive motor error *E*_*post*_. (5) Saccade peak velocity *κ*. (6) Saccade duration λ. Asterisks indicate significant difference between the pre- and post-exposure phase with *** *p* <.001, ** *p* <.01, * *p* <.05 and n.s. *p* ≥.05.

**Fig 6:**
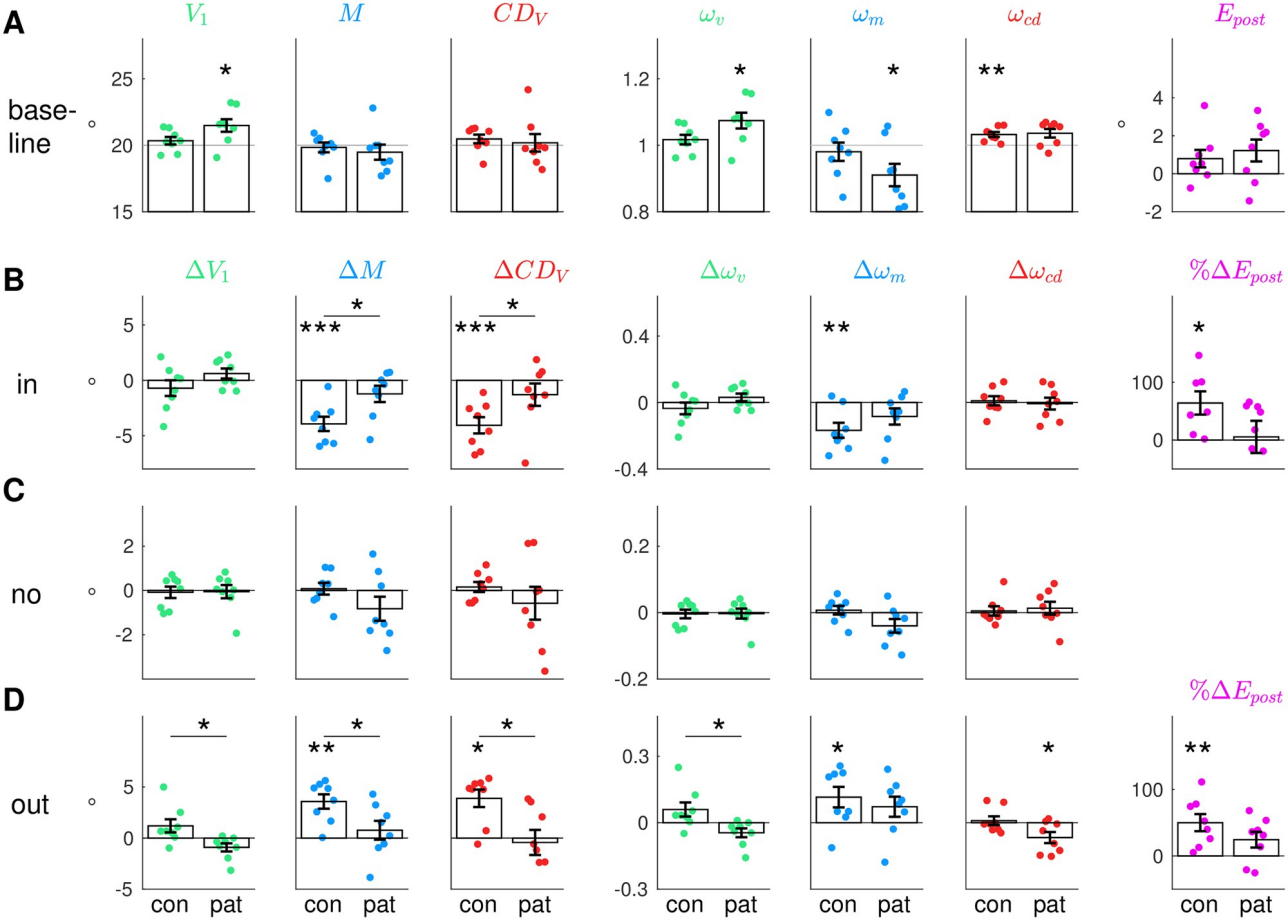
Baseline state and pre- to post-exposure changes. **(A)** Baseline state of the visuomotor system averaged across the pre-exposure phases of the three conditions (mean ± standard error), separately for control subjects and patients. Group-centered asterisks indicate significant difference from 20° (*V*_1_, *M*, *CD*_*V*_), from 1 (*ω*_*v*_, *ω*_*m*_, *ω*_*cd*_) or from zero (*E*_*post*_). **(B)** Pre- to post-exposure changes in the inward condition. **(C)** Pre- to post-exposure changes in the no step condition. **(D)** Pre- to post-exposure changes in the outward condition. For **B-D** group-centered asterisks indicate significant difference from zero. Panel-centered asterisks above a horizontal indicate significant difference between control subjects and patients with *** *p* <.001, ** *p* < .01, * *p* <. 05 and n.s. *p* ≥ .05.

**Fig 7:**
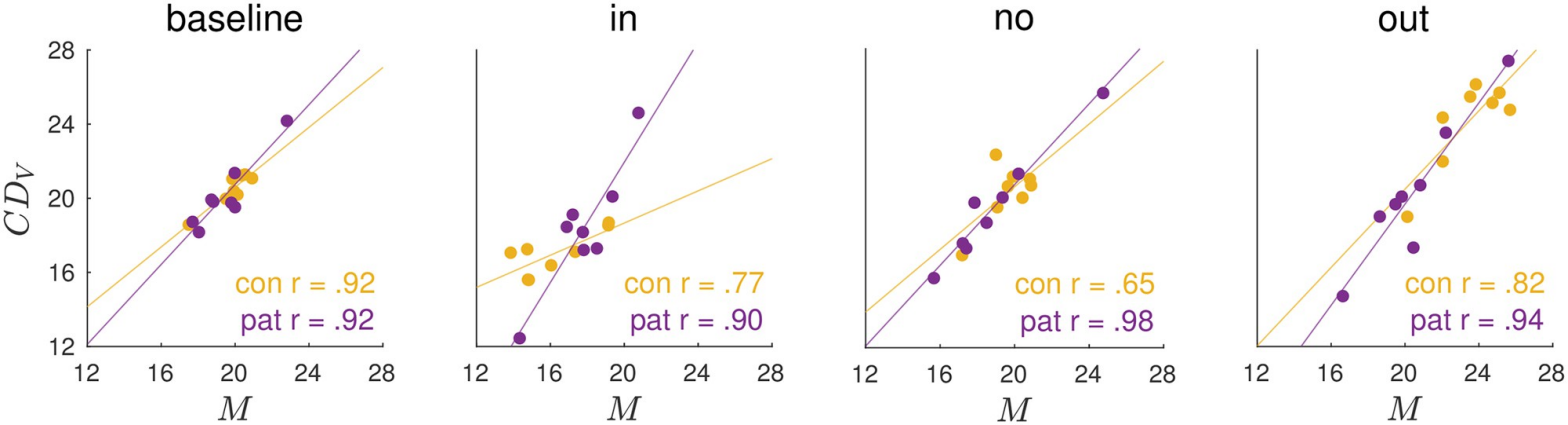
Correlation of motor command *M* and *CD*_*V*_ signal across subjects at steady state. At steady state, i.e. in the pre-exposure phase across all three conditions (baseline) and in the post-exposure phase of the inward condition, the no step condition and the outward condition, the motor command *M* and *CD*_*V*_ signal highly correlate across control subjects and patients. This shows that the quantification of the *CD*_*V*_ signal by the model based on the trans-saccadic target localization and saccade data is a valid estimate of the internal representation of the saccade.

**Fig 8:**
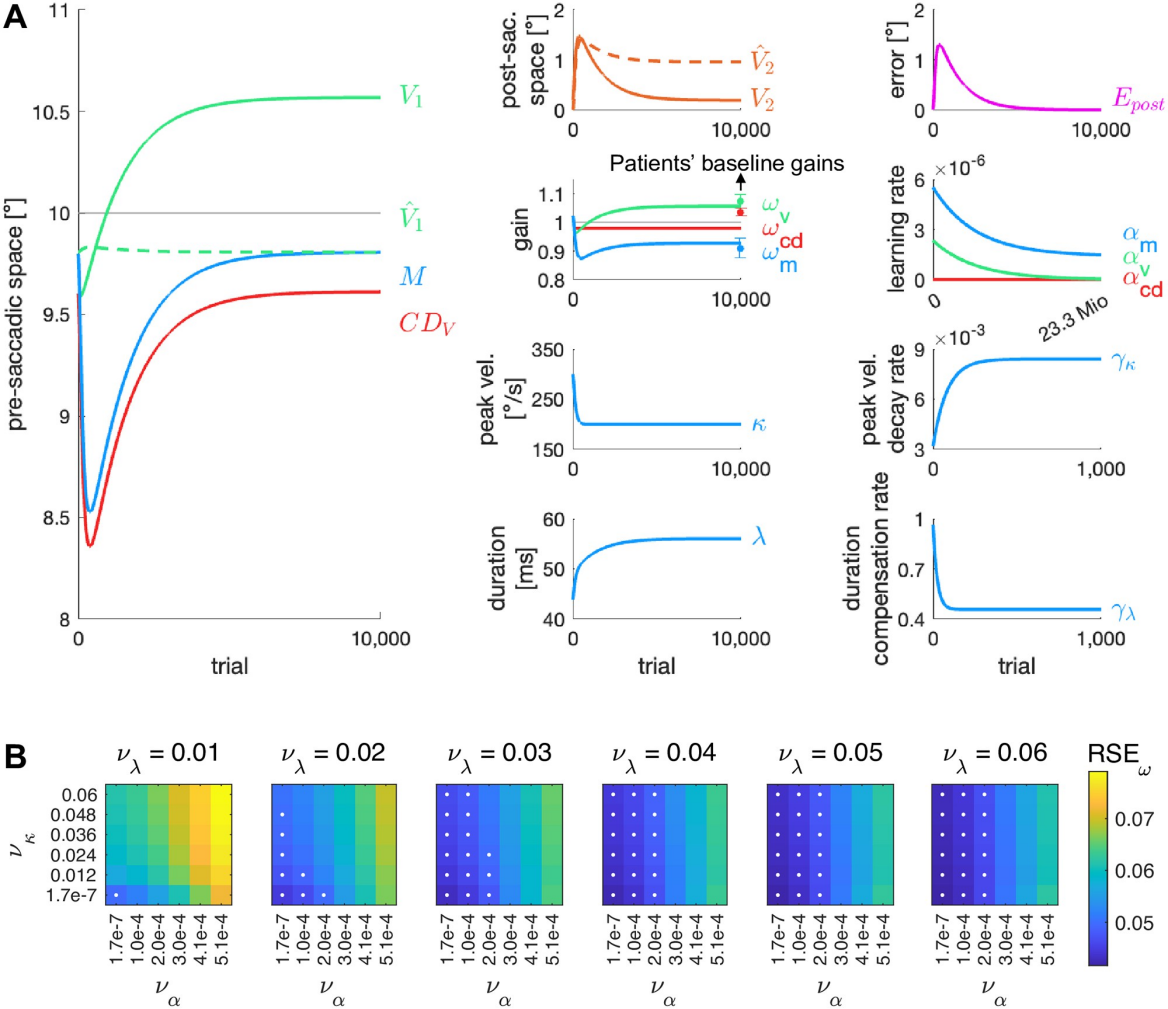
Simulation of long-term visuomotor behavior after disease onset. **(A)** Oculomotor fatigue in the very early phase of the disease causes a loss of saccade peak velocity *κ* that can only be partially compensated by upregulation of saccade duration λ. Consequently, a decline of the saccadic motor command *M* leads to saccade hypometry, that, in turn, results in a rise of motor errors *E*_*post*_. Preserved long-term learning at perceptual (*α*_*v*_) and motor level (*α*_*m*_)—that, yet, slowly decays throughout disease progression—counteracts to keep saccadic behavior calibrated. Hence, the visuomotor system stabilizes with an overestimation of the pre-saccadic target eccentricity (*V*_1_) and recovery from saccade hypometria (*M*), matching the baseline visuomotor gains measured in cerebellar patients (*ω*_*v*_ and *ω*_*m*_; *ω*_*cd*_ deviates; residual standard error *RSE*_*ω*_ = 0.05). Simulations started with the visuomotor gains *ω*_*v*_, *ω*_*m*_ and *ω*_*cd*_, the learning rates *α*_*v*_, *α*_*m*_ and *α*_*cd*_, the peak velocity decay rate *γ*_*κ*_ and the duration compensation rate *γ*_λ_ of healthy subjects; *α*_*v*_, *α*_*m*_, *α*_*cd*_, *γ*_*κ*_ and *γ*_λ_ change until they match the values of cerebellar patients. Progression rates were *ν*_*κ*_ = 0.012, *ν*_λ_ = 0.040 and *ν*_*α*_ = 1.7*10^−7^. Please note that the different subplots show different timescales. **(B)** Residual standard error *RSE*_*ω*_ depending on how fast learning rates (scaled by *ν*_*α*_), peak velocity decay rate (scaled by *ν*_*κ*_) and duration compensation rate (scaled by *ν*_λ_) change towards patients’ values. Simulations with *RSE*_*ω*_ ≤ 0.05 are marked with a white dot.

Fig 1B presents the framework of the expanded visuomotor learning model based on Masselink et al. (2021) [38] that we fitted to the saccade vectors and pre- and post-saccadic visual localizations, separately for each group (controls, patients) and each condition (inward, no step, outward). The visual pre-saccadic target *V*_1_ (fitted to the pre-saccadic visual localizations) is represented on the visuospatial map with the visual gain *ω*_*v*_ (Fig 1B-1). An inverse model transforms *V*_1_ into a motor command *M* (fitted to the saccade vectors) with the motor gain *ω*_*m*_ (Fig 1B-2). Before saccade execution, a copy of the motor command is routed into the CD pathway where a forward dynamics model estimates the visuospatial size *CD*_*V*_ of the upcoming saccade based on the CD gain *ω*_*cd*_ (Fig 1B-3). A forward outcome model then shifts retinal coordinates by *CD*_*V*_ to predict the retinal position of the post-saccadic target ${\hat{V}}_{2}$ (fitted to the post-saccadic visual localizations; Fig 1B-4). For saccade execution, the motor command is transposed to saccade peak velocity *κ* and saccade duration λ (Fig 1B-5). After the saccade is performed (Fig 1B-6), the actual post-saccadic target appears at retinal position *V*_2_ (Fig 1B-7). A backward outcome model then postdicts the visual post-saccadic target back to pre-saccadic space ${\hat{V}}_{1}$ (Fig 1B-8). The postdictive motor error *E*_*post*_ evaluates the accuracy of the motor command with respect to the postdicted target position in order to adapt its gains *ω*_*v*_, *ω*_*m*_ and *ω*_*cd*_ (Fig 1B-9). A decrease of *ω*_*m*_ (inward learning) is controlled by downregulating saccade peak velocity while an increase of *ω*_*m*_ (outward learning) is controlled by upregulation of saccade duration (Fig 1B-5).

The no step condition in which saccades are performed repetitively to the same, non-stepping target should lead to oculomotor fatigue, i.e. a decline in saccade peak velocity which is usually compensated by an increase of saccade duration. We captured peak velocity and duration changes in the no step condition by fitting a peak velocity decay rate *γ*_*κ*_ ≥ 0 and a duration compensation rate *γ*_λ_ (with 0 ≤ *γ*_λ_ ≤ 1). The latter describes the within-trial percentage by which saccade duration compensates for the peak velocity loss.

Fig 5 presents the model fits to the mean data of control subjects (Fig 5A) and patients (Fig 5B) for the three conditions (inward, no step, outward). Dots with errorbars show the mean data of the pre- and post-exposure phases, respectively, jagged blue lines show the trial-by-trial saccade data (Fig 5A-1 and 5B-1: saccade vectors; Fig 5A-5 and 5B-5: saccade peak velocities; Fig 5A-6 and 5B-6: saccade durations) and smooth lines show the trial-by-trial model fit to the data. Fig 6 depicts the baseline state as well as pre- to post-exposure changes for each condition, compared between patients and control subjects. *V*_1_ corresponds to the pre-saccadic target localization, *M* is equivalent to the measured saccade vector and *CD*_*V*_ relies on the interplay of post-saccadic target localization, pre-saccadic target localization and the measured saccade vector.

Table 2 gives an overview on the fitted parameters and residual standard errors as a measure of goodness of fit.

**Table 2:** Fitted parameters and residual standard errors.

Condition	Group	Fitted parameters	*β*_0_	*β*_*κ*_	*β*_λ_	$\underset{V_{1},M,{\hat{V}}_{2}}{RSE}$	$\underset{\kappa }{RSE}$	$\underset{\lambda }{RSE}$
Inward	Controls	*α*_*v*_ = 2.6*10^−6^, *α*_*m*_ = 2.1*10^−5^, *α*_*cd*_ = 5.9*10^−6^	-9.995	0.034	0.230	0.8°	25.0$\frac{{}^{{}^{\circ}}}{s}$	3.0 ms
	Patients	*α*_*v*_ = 0, *α*_*m*_ = 2.0*10^−6^, *α*_*cd*_ = 2.3*10^−13^	6.399	0.011	0.110	1.1°	49.8$\frac{{}^{{}^{\circ}}}{s}$	5.1 ms
No step	Controls	*γ*_*κ*_ = 0.003, *γ*_λ_ = 0.964	9.828	0.009	0.088	0.5°	23.7$\frac{{}^{{}^{\circ}}}{s}$	3.1 ms
	Patients	*γ*_*κ*_ = 0.008, *γ*_λ_ = 0.457	8.917	0.009	0.092	1.1°	56.1$\frac{{}^{{}^{\circ}}}{s}$	6.4 ms
Outward	Controls	*α*_*v*_ = 2.3*10^−6^, *α*_*m*_ = 5.5*10^−6^, *α*_*cd*_ = 5.3*10^−13^	-0.847	0.018	0.208	0.6°	21.8$\frac{{}^{{}^{\circ}}}{s}$	3.9 ms
	Patients	*α*_*v*_ = 0, *α*_*m*_ = 1.4*10^−6^, *α*_*cd*_ = 5.1*10^−7^	8.360	0.006	0.131	1.1°	50.8$\frac{{}^{{}^{\circ}}}{s}$	5.7 ms

Here we report the fitted parameters and residual standard errors for the goodness of model fit, separately for each group and condition. The learning rates *α*_*v*_, *α*_*m*_ and *α*_*cd*_ were fitted to the data of the inward and the outward condition, respectively. The peak velocity decay rate *γ*_*κ*_ and the duration compensation rate *γ*_λ_ were fitted to the data of the no step condition. The parameters *β*_0_, *β*_*κ*_ and *β*_λ_ are the weights of the regression from saccade vectors on saccade peak velocities and saccade durations. Residual standard errors are reported separately for saccade vectors and pre- and post-saccadic localizations ($RSE_{V_{1},M,{\hat{V}}_{2}}$), as well as for saccade peak velocities (*RSE*_*κ*_) and for saccade durations (*RSE*_λ_).

### Differences in learning of the visuospatial target map between patients and controls

The fit of the visual pre-saccadic target position *V*_1_ to the pre-saccadic target localizations (Fig 5A-1 and 5B-1, green) captures how the visuospatial target map, i.e. the visual gain *ω*_*v*_ (Fig 5A-3 and 5B-3, green), adapts on a short time-scale when errors are credited to an internal failure of visuospatial target representation. A small change of *V*_1_ in learning direction for control subjects could be observed, however this change did not reach significance (inward -0.7 ± 2.0°, *t*_7_ = -1.00, *p* = .351; outward 1.2 ± 1.8°, *t*_7_ = 1.86, *p* = .105; Fig 5A-1). As the change in pre-saccadic target localization across learning is a stable, substantial effect that, however, is usually rather small [24–27, 38, 57], it seems not surprising that we could not replicate a significant effect with a sample size of eight subjects. Please note that in the analysis of Cheviet et al. (2022) [48] (which comprised more trials including those in which the flash was presented with a small distance to the 20° target position), these changes were subtly significant. Accordingly, the change of the visual gain *ω*_*v*_ of control subjects was -0.04 ± 0.10 (*t*_7_ = -1.00, *p* = .351; Fig 6B) in the inward condition and 0.06 ± 0.09 (*t*_7_ = 1.86, *p* = .105; Fig 6D) in the outward condition.

The visual pre-saccadic target position *V*_1_ of cerebellar patients did not show any change in the direction of the target step. Small changes were observed in the opposite direction (inward 0.6 ± 1.3°, *t*_7_ = 1.35, *p* = .218; outward -0.9 ± 1.1°, *t*_7_ = -2.28, *p* = .057; Fig 6B and 6D) but were also not significant in the analysis of Cheviet et al. (2022) [48]. Adding a mechanism that allows the model to learn in the opposite direction of error reduction seemed not justified in this context. Accordingly, we fitted a stable *V*_1_ and a stable *ω*_*v*_ to the patients’ data (Fig 5B-1 and 5B-3, green). In sum, patients did not show short-term learning of the visual pre-saccadic target position *V*_1_ as compared to the tendency observed in control subjects. The *V*_1_ change was significantly different between patients and control subjects in the outward condition (*t*_14_ = 2.78, *p* = .015, Fig 6D) but failed to reach significance in the inward condition (*t*_14_ = -1.57, *p* = 0.138). This seems to rely on the higher adaptation level that control subjects reached in the outward condition compared to the inward condition. Differences in adaptation levels between target step directions have been reported before [12, 26, 58, 59].

As a tendency for a change of the visual gain in learning direction was observed in control subjects while remaining completely absent in patients, the cerebellum may play a role in the mediation of the visuospatial target map. However, it seems that a larger sample size is necessary to reliably examine whether cerebellar integrity plays a crucial role in this adaptive process.

### Cerebellar pathology reduces short-term learning of the inverse model

The fit of the motor command *M* to the saccade vectors (Fig 5A-1 an d 5B-1, blue) quantifies how the inverse model, i.e. the motor gain *ω*_*m*_ (Fig 5A-3 and 5B-3, blue), adapts when errors are assigned to false computation of the motor command to bring the eyes to the visuospatial target position. Hence, quantification of the motor gain change allows to evaluate inverse model adaptation in isolation, i.e. irrespective of the change of the visual pre-saccadic target representation *V*_1_ that is the input to the inverse model. In other words, the quantification of motor gain adaptation allows to differentiate to which cause errors are assigned to, i.e. to a false representation of the motor command *M* or to a false visuospatial representation of the target *V*_1_.

In accordance with previous studies [10, 11, 28, 38, 59–62], the motor command *M* of control subjects decreased by -3.9 ± 1.8° during inward learning (*t*_7_ = -6.06, *p* <.001; Fig 6B) and increased by 3.6 ± 2.0° during outward learning (*t*_7_ = 5.10, *p* = .001; Fig 6D). Accordingly, the inverse model, i.e. the motor gain *ω*_*m*_, adapted significantly in both learning conditions (inward -0.17 ± 0.13, *t*_7_ = -3.71, *p* = .008; outward 0.12 ± 0.13, *t*_7_ = 2.50, *p* = .041). As expected by the model, the motor command changes during inward learning were transposed to saccade kinematics by downregulation of saccadic peak velocity (-94.9 ± 46.2$\frac{{}^{{}^{\circ}}}{s}$, *t*_7_ = -5.81, *p* = .001; Figs 5A-5 and 3) while saccade duration stayed constant (-2.6 ± 7.3 ms, *t*_7_ = -1.01, *p* = .346; Figs 5A-6 and 4). By contrast, the motor command increase during outward learning was controlled by upregulation of saccade duration (10.4 ± 3.1 ms, *t*_7_ = 9.37, *p* <.001) while saccade peak velocity stayed constant (-11.0 ± 35.3$\frac{{}^{{}^{\circ}}}{s}$, *t*_7_ = -0.89, *p* = .405). The saccade changes brought control subjects’ eyes significantly closer to the post-saccadic target (*V*_2_ change by 3.9 ± 1.8° during inward learning, *t*_7_ = 6.06, *p* <.001; -3.6 ± 2.0° during outward learning, *t*_7_ = -5.1, *p* = .001; Fig 5A-2). Thus, control subjects successfully reduced the postdictive motor error *E*_*post*_ in both learning paradigms (inward 3.8 ± 2.4°, *t*_7_ = 4.53, *p* = .003; outward -3.3 ± 2.2°, *t*_7_ = -4.16, *p* = .004, Fig 5A-4). This corresponds to 64.1 ± 53.0% error reduction in the inward condition (*t*_6_ = 3.2, *p* = .019; Fig 6B) and 50.1 ± 36.3% error reduction in the outward condition (*t*_7_ = 3.9, *p* = .006; Fig 6D).

Compared to control subjects, motor command changes were largely reduced in cerebellar patients. During inward learning, the motor command *M* decreased by -1.2 ± 2.1° and increased during outward learning by 0.8 ± 2.6° (Fig 5B-1). These changes were not significant (inward *t*_7_ = -1.65, *p* = .143; outward *t*_7_ = 0.83, *p* = .433) and significantly less than in control subjects (inward *t*_14_ = -2.75, *p* = .016; outward *t*_14_ = 2.43, *p* = .029; Fig 6B and 6D). Accordingly, inverse model adaptation was not significant in patients (*ω*_*m*_ change inward -0.08 ± 0.14, *t*_7_ = -1.73, *p* = .127, outward 0.07 ± 0.13, *t*_7_ = 1.58, *p* = .158). Saccade peak velocity was significantly decreased in the inward condition (-55.9 ± 55.4$\frac{{}^{{}^{\circ}}}{s}$, *t*_7_ = -2.85, *p* = .025; Figs 5B-5 and 3) but saccade duration was not significantly increased in the outward condition (4.1 ± 13.7 ms, *t*_7_ = 0.84, *p* = .427; Figs 5B-6 and 4). Hence, reduction of postdictive motor error was less effective than in the control subjects, i.e. only 5.5 ± 78.8% during inward learning (*z* = 0.84, *p* = .401; Fig 6B) and 24.5 ± 33.4% during outward learning (*t*_7_ = 2.07, *p* = .077; Fig 6D). Reduction of *E*_*post*_ in the inward condition was significantly less in patients than in controls (*t*_14_ = 2.15, *p* = .049).

In sum, the motor gain adapted significantly in learning direction in control subjects but not in patients. This demonstrates that the cerebellum has a crucial role in the adaptation of the inverse model, i.e. when errors are assigned to a false computation of the motor command such that the saccade is erroneously scaled with respect to the visuospatial target position.

### Forward dynamics model is correctly informed about the reduced saccade change

Quantification of *CD*_*V*_ in our model (Fig 5A-1 and 5B-1, red) relies on the difference between pre- and post-saccadic localization, hence capturing *CD*_*V*_-based integration between the pre- and the post-saccadic visual scene (see Fig 5A-2 and 5B-2 for changes of the post-saccadic target localization with respect to saccade landing, i.e. ${\hat{V}}_{2}$). The CD gain *ω*_*cd*_ captures how the forward dynamics model is calibrated when errors are credited to a false internal representation of the saccade. When pre- and post-saccadic visual scene are perfectly integrated, i.e. when the flash is localized at the same position pre- and post-saccadically, *ω*_*cd*_ = 1 such that *CD*_*V*_ matches *M* and, hence, correctly reflects the performed saccade.

Fig 7 shows that the motor command *M* and the *CD*_*V*_ signal are highly correlated across control subjects and patients, respectively, in the pre-exposure phase (baseline state) as well as in the post-exposure phase of all three conditions (inward, no step, outward). This shows that the quantification of *CD*_*V*_ based on our paradigm is a valid estimate of the internal representation of the saccade.

In control subjects, the *CD*_*V*_ signal declined by -4.1 ± 2.1° during inward learning (*t*_7_ = -5.54, *p* <.001; Fig 6B) and increased by 3.9 ± 2.4° during outward learning (*z* = 2.38, *p* = .017; Fig 6D). Fig 5A-1 shows that *CD*_*V*_ well reflects the actual saccade changes in both learning paradigms. Thus, the initial CD gain (*ω*_*cd*_ = 1.03 ± 0.02, averaged as the baseline of all three conditions, Fig 6A) stayed stable throughout learning (inward 0.01 ± 0.08, *t*_7_ = 0.39, *p* = .707, Fig 6B; outward 0.01 ± 0.06, *z* = -0.42, *p* = .674, Fig 6D). This means that the forward dynamics model is well informed about the actual saccade changes in control subjects.

Surprisingly, this picture was similar in cerebellar patients. In both the inward and the outward condition, patients showed only a small change of the motor command *M* in learning direction that did not reach significance. This was well reflected by the *CD*_*V*_ signal. During inward learning, *CD*_*V*_ of patients was reduced by -1.3 ± 2.9° which did not reach significance (*t*_7_ = -1.28, *p* = .241, Fig 6B), thus well reflecting the actual saccade changes that were less than in control subjects (Fig 5B-1). In the outward condition, *CD*_*V*_ of patients stayed roughly constant (change of -0.4 ± 3.5°, *t*_7_ = -0.35, *p* = .737, Fig 6D). Accordingly, the initial CD gain (*ω*_*cd*_ = 1.04 ± 0.04, averaged as the baseline of all three conditions, Fig 6A) was not modified during inward learning (-0.01 ± 0.1, *t*_7_ = -0.19, *p* = .856) and only slightly modified during outward learning (-0.07 ± 0.07, *t*_7_ = -2.67, *p* = .032).

In sum, the forward dynamics model seemed correctly informed about the ongoing motor changes, similar in controls and patients (no difference in *ω*_*cd*_ change between patients and controls, inward *t*_14_ = 0.39, *p* = .704, outward *t*_14_ = 0.39, *p* = .704). This suggests that the forward dynamics model that is used for learning from post-saccadic errors remains unaffected by cerebellar disease.

### Cerebellar pathology is accompanied by overestimation of target eccentricity

Fig 6A presents the baseline state of control subjects and patients, averaged across the three conditions. While control subjects accurately localize the pre-saccadic target on the visual map (*V*_1_ = 20.3 ± 0.8°, t-test against *P*_1_ = 20°, *t*_7_ = 1.18, *p* = .276), patients showed a significant overestimation of target eccentricity by around 1.5° (*V*_1_ = 21.5 ± 1.3°, t-test against *P*_1_ = 20°, *t*_7_ = 3.13, *p* = .017). Hence, the visual gain *ω*_*v*_ overestimated the actual target distance already when patients entered the laboratory (*ω*_*v*_ = 1.07 ± 0.07, t-test against 1, *t*_7_ = 3.13, *p* = .017). This was consistent across all three conditions (Fig 5B-1). Still, patients exhibited a quite accurate baseline saccade (*M* = 19.5 ± 1.6°, t-test against *P*_1_ = 20°, *t*_7_ = -0.92, *p* = .389), i.e. the motor gain *ω*_*m*_ was downregulated such that the saccade was not hypermetric as expected from the overestimated target eccentricity (0.91 ± 0.1, t-test against 1, *t*_7_ = -2.66, *p* = .033). Interestingly, target overestimation despite a quite accurate saccade was observed before in a patient with a right posterior ventrolateral and right ventromedial thalamic lesion [63]. In spite of the visuospatial hypermetria, the patients’ visuomotor system seemed to be at a steady state as the postdictive motor error *E*_*post*_ did not significantly differ from zero (*t*_7_ = 2.11, *p* = .073 in patients, *t*_7_ = 1.73, *p* = .127 in control subjects, Fig 6A).

### Several patients tend to uncompensated oculomotor fatigue

The no step condition tested subjects’ ability to maintain a stable saccade vector when saccades were repetitively performed to the same, non-stepping target. Fig 5A-5 shows that control subjects underwent oculomotor fatigue, as revealed by the significant decline in saccade peak velocity by -45.7 ± 33.4$\frac{{}^{{}^{\circ}}}{s}$ (*t*_7_ = -3.87, *p* = .006, *γ*_*κ*_ = 0.003). However, peak velocity decline was counteracted by a significant upregulation of saccade duration (5.6 ± 5.4 ms, *t*_7_ = 2.90, *p* = .023) such that the saccade vector was maintained (*M* remained stable with *t*_7_ = 0.30, *p* = .775). The individual main sequences, i.e. the relationship of saccade peak velocity and saccade duration to the saccade vector, are shown in Figs 3 and 4. Fig 3 depicts that the reduction of saccade peak velocity in the no step condition is not accompanied by a reduction of the saccade vector in control subjects. Accordingly, Fig 4 depicts the increase of saccade duration while the saccade vector stays stable. With a duration compensation rate of *γ*_λ_ = 0.964, saccade duration compensated for 96.4% of the within-trial velocity loss. Thus, control subjects successfully kept the visuomotor system calibrated and the error nullified (Fig 5A-4).

The cerebellar patients group experienced a loss of peak velocity by -68.2 ± 102.2$\frac{{}^{{}^{\circ}}}{s}$ that, however, did not reach significance (*t*_7_ = -1.89, *p* = .101; *γ*_*κ*_ = 0.008). The high motor variability in conjunction with a small sample size of eight subjects make it difficult to draw clear conclusions from the patients’ data. Though, as the trend in peak velocity loss seemed reminiscent of the cerebellar patients of Golla et al. (2008) [42] (who observed reduced oculomotor fatigue compensation) and Xu-Wilson et al. (2009) [43] (who observed reduced within-saccade compensation for peak velocity fluctuations), we further investigated saccade peak velocity and saccade duration patterns in our sample. In our patients, upregulation of saccade duration could compensate only partially (7.7 ± 14.9 ms, *t*_7_ = 1.46, *p* = .187) such that the saccade vector declined by -0.8 ± 1.5° (*t*_7_ = -1.52, *p* = .172). Please note that also here, the patients’ data were highly variable and both effects did not reach significance at group level. Fig 6C shows that the saccade fell short in five of eight patients (saccade change from the pre- to the post-exposure phase of patient P1 -1.8°, P3 -1.4°, P6 -1.5°, P7 -2.7° and P8 -1.9°). This tendency is spared in patients P2, P4 and P5 (saccade change from the pre- to the post-exposure phase of patient P2 1.6°, P4 0.9° and P5 0.2°). Only one subject of the control group showed a comparable decline of -1.2° (subject C8; control subjects C1-C7 showed a saccade change of -0.4°, -0.3°, -0.1°, 0.2°, 0.3°, 1.0° and 1.0°). The individual main sequences of patients can be seen in Figs 3 and 4 including disease type and disease duration of each patient. Two of three patients with cerebellar ataxia (patients P1 and P3 but not patient P5) and one of two patients with Friedreich ataxia (patient P8 but not patient P2) show the impairment. Neither the disease type nor the specific anatomical involvements of the disease (Table 1) seem to be related to the impairment.

The fit of the saccade duration compensation parameter *γ*_λ_ to the patients’ data suggests that patients could compensate only 45.7% of the within-trial peak velocity loss (*γ*_λ_ = 0.457), resulting in uncompensated oculomotor fatigue (Fig 5B-1). Still, due to the lack of significance at group level, this result describes a tendency that needs further examination. Anyhow, the suggested deficit in saccade duration control during oculomotor fatigue appears consistent with the greater impairment during outward learning in which changes of the inverse model are usually transposed to an increase of saccade duration (Fig 5B-1 outward condition).

### Overestimation of target eccentricity may be a result of error reduction to counteract uncompensated oculomotor fatigue

We wondered how the patients’ tendency for uncompensated oculomotor fatigue affected their natural visuomotor behavior in the long run. Saccade kinematics underlie continuous fluctuations and if saccade duration cannot sufficiently compensate for regular declines of saccade peak velocity, the visuomotor system will inevitably tend to be hypometric. Saccade hypometry has been observed as a consequence of vermal cerebellar lesion in monkeys [39, 40, 64, 65] and in humans [66]. However, Takagi et al. (1998) [39] and Barash et al. (1999) [40] reported that saccade vectors recovered within weeks to months. Notably, baseline saccades of cerebellar patients in our experiments were not hypometric (19.5 ± 1.6°, *t*_7_ = -0.92, *p* = .389) and not different from those of control subjects (*t*_14_ = 0.30, *p* = .767). Thus, if hypothetically, saccade hypometria occurred in the early phase of the disease, the visuomotor system would necessarily have experienced significant motor errors due to the saccade vector decline. Even if short-term error reduction is reduced in cerebellar patients, a certain ability for long-term learning is assumed to be preserved [40, 43, 56, 67]. Hence, we simulated how the visuomotor system will behave on a long time course, starting with the visuomotor gains *ω*_*v*_, *ω*_*m*_, *ω*_*cd*_, the learning rates *α*_*v*_, *α*_*m*_, *α*_*cd*_, the peak velocity decay rate *γ*_*κ*_ and the duration compensation rate *γ*_λ_ of healthy control subjects. Through the course of the simulation, the learning rates *α*_*v*_, *α*_*m*_, *α*_*cd*_, the peak velocity decay rate *γ*_*κ*_ and the duration compensation rate *γ*_λ_ successively changed towards the respective values obtained from the patients’ model fit.

Fig 8A shows a simulation for 23,3 Mio saccades directed towards a 10° goal. We chose the initial visuomotor gains *ω*_*v*_, *ω*_*m*_ and *ω*_*cd*_ measured for similarly sized saccades in 35 healthy subjects by Masselink et al. (2021) [38] because the larger sample size of Masselink et al. (2021) provides a more faithful picture than the initial gains of the healthy group of Cheviet et al. (2022) [48]. The simulation shows that a loss in saccade peak velocity and a reduced saccade duration compensation in the very early phase of the disease leads to an immediate decline of the motor command *M*, hence shortening the saccade vector from 9.8° to 8.5°. In turn, the visuomotor system starts to counteract the growing motor error *E*_*post*_, inducing outward learning at perceptual (*ω*_*v*_) and motor level (*ω*_*m*_). Consequently, learning to counteract oculomotor fatigue leads to an outward shift of the visual pre-saccadic target localization and simultaneous recovery from saccade hypometry. The steady state at which the visuomotor system arrives is consistent with the baseline gains that we measured when patients came to the laboratory (residual standard error *RSE*_*ω*_ = 0.05). The simulation depicts a very fast progression of the peak velocity decay rate (scaled by *ν*_*κ*_ = 0.012) and the duration compensation rate (scaled by *ν*_λ_ = 0.040) towards patients’ values but the learning rates change very slowly across 23,3 Mio saccades (scaled by *ν*_*α*_ = 1.7*10^−7^). Please note that most signals are shown across shorter timescales because they converge early.

We examined how fast the parameters can change towards patients’ values such that the simulation converges on the patients’ baseline visuomotor gains including outward localization of visual targets. Fig 8B depicts the residual standard error of the visuomotor gains at the end of the simulation (*RSE*_*ω*_) depending on how fast learning rates, peak velocity decay rate and duration compensation rate change towards patients’ values (scaled by the parameters *ν*_*α*_, *ν*_*κ*_ and *ν*_λ_). A white dot marks the simulations with *RSE*_*ω*_ ≤ 0.05 (reflecting a mean gain deviation below 4% at the end of the simulation). The simulation can converge on the patients’ baseline visuomotor gains, and, hence, reflect the visual outward localization that we found in the patients, if oculomotor fatigue compensation impairment occurs in the very early phase of the disease while learning is almost still intact, i.e. the learning rates decay slowly towards patients’ values. Only then, preserved learning can counteract the saccade vector decline.

The depicted timescale in Fig 8A would be much longer in a natural real life situation as saccades are usually performed to goals of different directions and eccentricities. Hence, our simulation is a simplification of the changes in oculomotor control during cerebellar disease progression and rather suggests relative timescale differences between the changes in peak velocity decay, duration compensation and learning rates. Moreover, the simulation remains speculative as long as we cannot compare the simulated disease progression to long-term data of cerebellar patients. However, we suggest that intertemporal oculomotor fatigue of multiple occurrence could lead to gradual changes of the visuomotor system similar to our simulation in the long run. Hence, the overestimation of target eccentricity in cerebellar patients may be a consequence of long-term perceptual learning to reduce motor errors.

## Discussion

To dissociate cerebellar contributions to three stages of error-based learning, we modeled the visuomotor learning data of eight patients with a neurodegenerative cerebellar disease and eight healthy control subjects. The model, based on Masselink et al. (2021) [38], captures recalibration (1) of the visuospatial target map, (2) of an inverse model that transforms the visuospatial movement goal into a motor command, and (3) of a forward dynamics model that estimates the saccade size from corollary discharge. The model learns from postdictive motor error, i.e. motor error with respect to a postdictive update of the pre-saccadic target position. In patients, short-term learning was reduced in the inverse model and abolished in the visuospatial target map while the forward dynamics model seemed correctly informed about the reduced saccade change. Moreover, some types of cerebellar pathology impaired the ability to upregulate saccade duration in response to oculomotor fatigue. Modeling suggests that long-term error reduction at perceptual level may have counteracted, explaining the overestimation of target eccentricity in the patients’ baseline data. We conclude that the cerebellum recalibrates the inverse model on a short time-scale, particularly via control of saccade duration, while the forward dynamics model does not depend on cerebellar integrity. Long-term perceptual learning may partially compensate for cerebellar motor deficits.

### Cerebellum implements short-term learning of the inverse model

Our results confirmed that the inverse model is adapted within the cerebellar microcircuit. Motor gain adaptation was largely restrained in cerebellar patients for both inward and outward errors. This means that the cerebellum is specifically involved in the part of adaptation that relies on credit assignment to a false computation of the motor command, i.e. irrespective of the adaptation of the visual pre-saccadic target representation. Similar impairments have been shown in lesioned monkeys [39, 40], in cerebellar patients [41–43] and in healthy subjects with tDCS or magnetic stimulation of the cerebellum [45, 46, 68]. In the patients of Golla et al. (2008) [42], learning from outward target steps was completely suspended while in our study, a small saccade change was preserved. This difference may arise because the patients’ lesion of Golla et al. (2008) [42] was specifically located in the oculomotor vermis, i.e. the particular region assumed to process inverse model adaptation for oculomotor commands [56, 69, 70].

### Cerebellar role in short-term learning of the visuospatial target map requires further examination

After visuomotor learning has initially been seen through a pure motor lens, numerous studies have revealed changes in the visuospatial perception of the movement goal [24, 25, 27]. Hence, motor learning is not simply a change of the motor command directed to the same goal location on every trial. Instead, learning underlies recalibration of both the visuospatial goal representation and the motor command required to bring the eyes to that location. This makes sense since motor errors cannot only be due to erroneous computation of the motor command but can also stem from a deficient goal representation. Control subjects showed a learning trend in the visual target representation, yet, in contrast to previous studies [24–27, 38, 57] this change did not reach significance, probably due to a small sample size. In patients, a learning trend was not observable. Hence, evidence of differences between patients and control subjects is still lacking and further examination is required to evaluate whether the cerebellum is a neural substrate for visuospatial recalibration during motor learning. Neurophysiologically, the cerebellum is an ideal candidate to modulate visuospatial plasticity because of its inner circular organization [70, 71], its homogenous output organization [17, 18, 72], and its feedback projections to cortical retinotopic areas [19–21].

### The forward dynamics model is not affected by cerebellar pathology

Consistent with previous work [24, 25, 28, 38], in our study, healthy subjects’ localizations shifted pre- and post-saccadically while both changes remained absent in patients. Thus, *CD*_*V*_ seemed to correctly reflect the saccade changes in healthy controls and in patients. This result has two major implications. First, it proposes that the efference copy that is routed into the forward dynamics model reflects the actual adapted motor command and not some kind of initial motor command prior to adaptive changes. Second, it suggests that also in patients, the forward dynamics model accurately captures the actual changes of the inverse model such that the saccade vector is neither over- nor underestimated. This means that the forward dynamics model involved in trans-saccadic spatial integration and feedback motor learning remains unaffected by cerebellar degeneration. Indeed, the cerebellum computes forward models for object motion [73–75] and feedforward motor control (movement adjustments during execution [53, 62, 76]). However, the forward dynamics model measured in our task is one involved in trans-saccadic perception and feedback motor learning, i.e. from errors after movement completion. That this forward dynamics model may rely on areas upstream of the cerebellum is supported by studies showing that a disruption of the CD pathway from SC via MD thalamus to the FEF provokes a bias in visual space updating [77–80]. The forward dynamics model could also be computed along other CD pathways, e.g. from SC via the thalamic pulvinar to parietal and occipital cortex [81, 82]. The role of parietal cortex in CD processing was recently demonstrated by Cheviet et al. (2022) [48]. At least, saccadic learning involves a distributed network across cerebral cortex [83–85].

### Cerebellar role in oculomotor fatigue compensation

Our results motivate further examination of whether cerebellar integrity may be crucial for counteracting oculomotor fatigue by saccade duration adjustment. While healthy subjects compensated peak velocity loss by 96% within a trial, patients achieved only 46%. As peak velocity loss and saccade duration compensation did not reach significance at the patients’ group level, the interpretation of these results need to be treated with caution. Still, the observed tendencies are in line with previous data of patients whose disease affected the oculomotor vermis. Firstly, Golla et al. (2008) [42] observed reduced oculomotor fatigue compensation, and, secondly, Xu-Wilson et al. (2009) [43] observed reduced within-saccade compensation for peak velocity fluctuations. Taken together, it seems worth to further examine whether saccade lengthening, within-saccade (due to oculomotor fatigue) or after saccade (due to post-saccadic motor error), is operated by cerebellar-dependent saccade duration control. That the cerebellum accommodates a velocity-duration tradeoff has been shown for hand pointing movements by Markanday et al. (2018) [86]. While movement duration was fine-tuned with respect to movement velocity in healthy controls, the velocity-duration tradeoff was completely absent in patients with different types of global cerebellar degeneration, i.e. comparable to our patient sample. It has to be noted that in our sample, the saccade fell short throughout the no step condition in five of eight patients with different types of cerebellar disease while only one control subject showed a comparable decline. Further examination of the role of the disease type could help to understand which cerebellar structures are critical to adjust saccade duration during oculomotor fatigue.

The cerebellum adjusts movement duration in response to velocity variations also for smooth pursuit [87]. Neurophysiologically, it remains to be examined how duration control is implemented. For manual movements, there is first evidence that the simple spikes of Purkinje cells also code movement velocity and duration [88–90].

### Long-term perceptual learning may compensate for cerebellar deficiencies in saccade duration control

In the long run, insufficient velocity compensation in cerebellar patients will lead to hypometric saccades. Indeed, saccade hypometry and reduced saccade velocity were observed in cerebellar patients [42, 66] and lesioned monkeys [39, 40, 64, 65]. Even if the affected part of the cerebellum in these studies was restricted to the oculomotor vermis, we also found a hypometric tendency in the course of repetitive saccades in some patients. Our model simulation for long-term behavior of a cerebellar-deficient visuomotor system suggests how saccade hypometry may occur and, with the gradual increase of motor errors, will be counteracted by learning at perceptual and motor level. The simulation can explain the significant overestimation of target eccentricity in the patients’ baseline data on the condition that the impairment in oculomotor fatigue compensation occurs in the very early phase of the disease while learning impairments proceed very slowly, and, hence, become apparent in the later phase of the disease.

Moreover, the simulation depicts a recovery from saccade hypometry as found in monkeys with cerebellar lesion [39, 40]. Yet, unlike the progressive nature of the cerebellar diseases in our patient sample, the cerebellar lesions in these studies occurred abruptly. In addition, recovery concerned saccade accuracy but not saccade precision, like in our patients who exhibited non-hypometric saccades despite high endpoint variability in the baseline. Hence, a tendency for saccade dysmetria due to deficiencies in saccade duration control may induce long-term perceptual and motor learning, probably upstream of the cerebellum. This may enable the visuomotor system to stay calibrated despite cerebellar short-term adaptation deficits.

Neurophysiologically, the visual overestimation of target eccentricity is interesting when put together with Zimmermann et al. (2015) [63] who found a similar result in a patient with a right posterior ventrolateral and ventromedial thalamic lesion. If, in both cases, this visual peculiarity is a consequence of motor adaptation deficits, these results underline the critical role of the cerebellar projection pathway via VL thalamus to frontal cortex for intact motor learning [47, 91].

### Implementation of the postdictive motor error in the cerebellum

To model visuomotor behavior we used a postdictive error signal, i.e. the postdictve motor error, that drives learning of the pre-saccadic visual target position, the motor command and the *CD*_*V*_ signal. Previous models on oculomotor learning a) have solely been focused on adaptation of the saccade vector and b) were usually driven by visual prediction error, thereby assuming that the saccade is always predicted to land with a stable target undershot as measured in the baseline [53, 92]. In Masselink et al. (2021) [38] we developed a new paradigm that allowed us to estimate the internal saccade size from pre- and trans-saccadic target localization with respect to the actually performed saccade, showing that this assumption cannot hold. While visual prediction error could not explain learning, learning was well explained by postdictive motor error. According to this, the visuomotor system computes a postdictive update of the pre-saccadic target position based on post-saccadic visual input and derives the error of the executed motor command with respect to this position. Supporting this idea, we recently found that adaptive changes to saccade targeting can be induced by post-saccadic visual feedback alone, i.e. when a pre-saccadic target is never shown [93]. It remains to be examined how such a postdictive error signal may be implemented in the cerebellum. The complex spikes of cerebellar Purkinje cells code visual and motor events, including the eccentricity of the post-saccadic target that is measurable 50 to 250 ms after saccade landing [7]. Post-saccadic target eccentricity could be merged with *CD*_*V*_ information carried by the parallel fibers [4, 5, 94] to form the postdictive update of the target that is used to evaluate the motor command.

### Limitations

Our model provides insights into how learning of visuomotor gains and oculomotor fatigue compensation is impaired in cerebellar patients. However, our model does not explain the increased within-subject and cross-subject saccade variability of the patients compared to the healthy control subjects. Increased within-subject variability of saccade endpoints is known from previous studies on cerebellar pathology [39, 40, 42, 43, 56]. The source of this phenomenon is not resolved yet and its effect on saccade motor learning remains to be examined. However, increased endpoint variability seems consistent with the role of the cerebellum in the within-saccade adjustment of movement duration in response to velocity changes that we found in the no step condition (see also Xu-Wilson et al., 2009 [43]). If the cerebellum cannot sufficiently adjust movement duration, i.e. on the basis of feedforward saccade control [43, 53, 62, 76], saccade endpoints will not only tend to be hypometric (in case of overall velocity decline across saccades) but will additionally be more variable as a result of trial-to-trial velocity fluctuations.

The increased cross-subject saccade variability of cerebellar patients may be due to the different types of cerebellar disease. However, we could not determine a relationship between disease type and saccadic behavior in our patient sample, e.g. by detecting systematic similarities of saccadic behavior between the three patients with spinocerebellar ataxia or between the two patients with Friedreich ataxia. As patients are difficult to recruit for study participation, the sample size was limited to eight patients and eight healthy control subjects. A larger sample size and a systematic analysis of similarities and differences in saccadic behavior is required to further examine how impairments of visuomotor learning and oculomotor fatigue compensation differ depending on the specific disease type and affected cerebellar areas.

In our model, we fit the pre-saccadic visual target position to visual localizations performed with a mouse cursor. The pre-saccadic visual target position defines the spatial saccade goal and is used as the input to the inverse model. The inverse model computes the muscle dynamics in order to bring the eyes to the target. Fitting the pre-saccadic visual target position to the visual localization data implies that the explicitly reported perception of the target is equivalent to the implicit visual representation that is used as a spatial saccade goal by the oculomotor system. The quantification of inverse model adaptation (that is derived from saccade changes relative to pre-saccadic localization changes) is sensitive to this assumption. However, consistent with this assumption, it seems plausible that pre-saccadic localization changes during learning [23–27, 38, 57] reflect a change of the spatial saccade goal and not a sole change of visual perception without any effect on saccade execution. The reason lies within the nature of the task itself that serves a motor goal, i.e. to perform accurate movements, not to perform accurate perception. Learning is an optimization problem, and hence, should serve the optimization of the task goal. A sole change of target perception without any effect on saccade execution would not optimize the task goal, i.e. it would not optimize motor function, and, thus, would be useless. Hence, the presence of pre-saccadic localization changes during learning argue for a use by the motor system, i.e. as a spatial movement goal.

Current models of motor learning often include a decay rate that pulls behavior back to a baseline level, thereby introducing a trace of motor memory [92, 95–98]. This allows to explain possible short-term forgetting of the learned behavior, e.g. during set breaks, that our model does not provide. In this approach, forgetting relies on the assumption that learned motor behavior is represented as a deviation from a baseline state of visuomotor transformations that is ingrained from the millions of saccades made in natural conditions and stays stable over the time of a typical adaptation experiment [99]. Our model differs from these models in that it is not based on a deviation from a steady state. In our model, the visuomotor system stays in a steady state that is reached, and can flexibly transit to a next steady state when another perturbation is applied without a pull-back process to a previous steady state. This still allows the model to return to baseline in case a perturbation is canceled because then, errors indicate that motor performance needs to be optimized by adaptation in the opposing direction. It would be interesting for future work to see how long-term learning and short-term adaptation can be combined in our model, perhaps by including multiple parallel learning processes at different time scales.

### Conclusion

We suggest an expanded view on the cerebellar circuit function for oculomotor learning and fatigue compensation. The cerebellum does adapt the inverse model but the forward dynamics model for trans-saccadic space perception and feedback motor learning may be processed upstream of the cerebellum. Future research needs to examine the cerebellar role in visuospatial recalibration, and where the forward dynamics model is computed, e.g. in the thalamus, in the frontal eye fields or in other cerebral areas.

## Methods

### Subjects

Our study is based on the dataset of Cheviet et al. (2022) [48] which includes eight healthy control subjects (49.25 ± 7.67 years, four male, four female) and eight patients with a neurodegenerative disorder of the cerebellum (55.5 ± 9.61 years, four male, four female). Please see Table 1 listing the individual patients’ characteristics.

Each patient was clinically examined at the Neurological Hospital Pierre Wertheimer (Bron, France) to verify a chronic progressive cerebellar ataxia, normal or corrected-to-normal vision as well as the ability to concentrate and to remain seated for at least 30 min. Another inclusion criterion was that patients were able to maintain a stable hand position for at least 30 s. This ensured that visual target localizations performed with a mouse cursor were not affected by tremor. Patients with another neurological disease, unstable medical condition, psychotropic medication intake, pronounced nystagmus or ocular instability were excluded from study participation.

### Ethics statement

All subjects gave written informed consent to participation. The experiment was performed in accordance with the ethical standards of the 1964 Helsinki Declaration and was approved by the ethics committee (Comité de Protection des Personnes Est-III; ID-RCB: 2017–00942-51; 17.05.09).

### Setup

Subjects were seated in a completely dark room in front of a CRT monitor (19 inches, 1280 × 1024 pixels, 85 Hz, 57 cm viewing distance) that was covered with a neutral density filter to prevent visibility of monitor background light and contrast to the surrounding (ND4, 25% transmittance, 0.03 $\frac{cd}{m^{2}}$ stimuli luminance, 0.01 $\frac{cd}{m^{2}}$ room luminance).

Movements of the right eye were recorded by an Eyelink 1000 (remote configuration; SR Research, Ontario, Canada) at 1000 Hz with a five-point calibration before each session and drift correction after each session break. Online fixation control was based on a 5° position threshold (horizontally and vertically). Saccade onset was detected online by a 2.5° position threshold, a 22$\frac{{}^{{}^{\circ}}}{s}$ velocity threshold and a 4000$\frac{{}^{{}^{\circ}}}{s^{2}}$ acceleration threshold. With respect to saccade initiation (0 ms), in the cerebellar group, the saccade was detected online at 16.1 ± 2.3 ms, the target was stepped (in saccade trials) or disappeared (in post-saccadic localization trials) at 28.9 ± 2.3 ms while the saccade ended at 72.7 ± 23.2 ms. In the healthy group, from saccade initiation (0 ms), the saccade was detected online at 15.5 ± 1.6 ms, the target was stepped (or disappeared) at 28.3 ± 1.5 ms while the saccade ended at 64.1 ± 3.8 ms. The procedure was controlled with Experiment Builder (SR Research, Ontario, Canada).

### Experimental design

Each subject participated in three experimental conditions recorded in randomized order with at least one week in between. Each condition measured reactive saccades to a 20° rightward target as well as visuospatial localizations of a flashed bar that varied horizontally around the target position by -4, -2, 0, +2 and +4° (Fig 1A). Localization was performed during fixation (pre-saccadic localization trials) and after saccade landing (post-saccadic localization trials).

The pre-exposure phase measured subjects’ baseline state, comprising four repetitions of five pre-saccadic localization trials and five post-saccadic localization trials, followed by a block of 20 saccade trials with peri-saccadic target offset (4*(5+5) + 20 = 60 trials; for an overview of the trial sequence see Fig 2A-B). The exposure phase measured 120 saccade trials with either a 6° peri-saccadic target step opposite to saccade direction (inward condition), or a 6° peri-saccadic target step in saccade direction (outward condition), or without any peri-saccadic target step (no step condition). The relatively large target step of 6° was chosen because cerebellar dysfunction is usually accompanied by a strong variability of saccade amplitudes, thereby leading to a strong variability of the post-saccadic visual target position [39, 40, 42, 43, 56]. In order to keep this disturbance as small as possible, we chose a rather large target step, and, consequently, a rather large pre-saccadic target eccentricity of 20° such the target step was 30% of the pre-saccadic target eccentricity. Control subjects and patients reported that they did not notice the target step. In contrast to the two learning conditions, the no step condition required subjects to maintain the saccade vector, which is usually achieved by upregulating saccade duration to counteract peak velocity decline in the course of repetitive, stereotyped saccades, known as oculomotor fatigue. A break was performed after the 50th and the 100th trial.

The post-exposure phase measured localizations after exposure (four repetitions of five pre-saccadic localization trials and five post-saccadic localization trials), interleaved with three blocks of each 20 saccade refresh trials (including the respective target step) to maintain the achieved saccade vector of the exposure phase (4*(5+5) + 3*20 = 100 trials; Fig 2A-B). Each session began with a 10 min dark adaptation phase to ensure that subjects were able to discriminate the stimuli with low luminance already at the start of the session. During session breaks (before each change of trial type and after the 50th and 100th trial of the exposure phase), the room was dimly illuminated to keep the level of dark adaptation constant. The Eyelink illuminator was not visible to the participants, even after dark adaptation because the 940-nm model was used.

All stimuli (target, flash and pointer) were presented with respect to the average horizontal gaze position of a 50 ms fixation reference period at trial start (fixation reference, see trial descriptions below). This procedure ensured consistent stimulus eccentricity despite reduced fixation accuracy of cerebellar patients. A trial was repeated if fixation criteria were violated. Inter-trial interval was a 800 ms black screen.

#### Saccade trials

A red fixation circle of 1° diameter appeared for a random time interval between 800 and 1400 ms (5° to the right of the left screen border, vertically randomized between -1.5, 0 and 1.5° to the horizontal meridian, Fig 1A). If fixation criteria were met during this period, a red target circle was displayed 20° to the right with respect to the fixation reference determined during the last 50 ms of the fixation period. As soon as saccade onset was detected, the target switched off in the pre-exposure phase, and, in the exposure and post-exposure phase, stepped either 6° inward (inward condition), 6° outward (outward condition) or stayed at its position (no step condition). In the exposure and post-exposure phase, the target stayed visible for 100 ms.

#### Post-saccadic localization trials

A red fixation circle of 1° diameter was presented 5° to the right of the left screen border on the horizontal meridian (Fig 1A). Subjects were instructed to fixate the circle and to indicate when ready to begin the trial via mouse press. If, after a 450 ms black screen, fixation criteria were met for 50 ms, a blue vertical line was flashed with counterbalanced horizontal distance of 16, 18, 20, 22 or 24° with respect to the fixation reference determined during the 50 ms fixation check period (12 ms, 0.2° width, 29° height). After a 400 ms black screen, a red target circle of 1° diameter appeared 20° to the right of the fixation reference. As soon as saccade onset was detected, the target was extinguished, followed by a 100 ms black screen. A blue line pointer was displayed on the right screen bottom (0.2° width, 10° height, 10° below the horizontal meridian, randomly 17, 19, 21 or 23° to the right of the fixation reference). As soon as subjects started moving the mouse, the pointer was blocked to the horizontal meridian. The subject had to move the pointer to the perceived horizontal flash position, confirming the judgement with a mouse press.

Please note that in case of any dysmetria of arm movements, patients had enough time to adjust the cursor position until it visually matched the position where they had perceived the flashed vertical line. As all patients were able to hold a stable position of the hand for at least 30 s, the mouse click could not be affected by tremor and should solely rely on the patients’ perceived position of the flashed line.

#### Pre-saccadic localization trials

A red prohibited direction sign served as a fixation stimulus (with the same spatial parameters as in the post-saccadic localization trials, Fig 1A). The subject had to continuously fixate it from the first mouse press (to indicate she/he is ready to start the trial) to the second mouse press (to confirm the localization judgement). The flash was presented with the same latency and spatial parameters as in the post-saccadic localization trials. The pointer appeared 750 ms after flash offset to roughly match the respective interval of the post-saccadic localization trials, and with the same latency and spatial parameters as in the post-saccadic localization trials. As soon as the subject started moving the mouse, the pointer was blocked to the horizontal meridian and the subject had to move the pointer to the perceived horizontal flash position and press the mouse.

#### Training phase

Before each session, subjects practiced a minimum of 5 pre- and 5 post-saccadic localization trials. In case of difficulties, online gaze position and a fixation box around the fixation circle were displayed to help subjects to familiarize with the task requirements.

### Model

To quantify the adaptive changes in the visuomotor circuitry of patients and control subjects, we used the model of Masselink et al. (2021) [38] and expanded it with (1) how changes in the motor command are transposed to saccade peak velocity and saccade duration and (2) how the visuomotor system compensates for a fatigue-induced decline in saccade peak velocity (Fig 1B).

#### Model conception

The model describes the saccadic circuitry based on three sensorimotor transformations, i.e. synaptic gains [14, 15]. These plastic gains are, first, a visual gain *ω*_*v*_ to transform retinal input into target position on a visuospatial map, second, a motor gain *ω*_*m*_ to transform spatial target position into a motor command (inverse model), and, third, a CD gain *ω*_*cd*_ to transform the corollary discharge *CD*_*M*_ of the motor command into *CD*_*V*_, i.e. the computed displacement of visual space due to the saccade (forward dynamics model).

The three gains learn to reduce postdictive motor error, an error of the motor command with respect to a postdictive update of the pre-saccadic target position. We use the postdictive motor error for learning based on the results of Masselink et al. (2021) [38]. Previous models on oculomotor learning have solely been focused on adaptation of the saccade vector, learning either from post-saccadic visual error or from visual prediction error [53, 62, 76, 100]. What argues against the post-saccadic visual error is that the visuomotor system accepts a remaining amount of post-saccadic visual error when learning reaches a steady state, i.e. when it reaches saturation [101–103]. This means that post-saccadic visual error is not nullified at steady state. Moreover, the use of visual prediction error is usually based on the assumption that the saccade is predicted to land with a stable target undershoot as measured in the baseline. In Masselink et al. (2021) [38], we quantified the prediction, i.e. the internal visuospatial representation of saccade size *CD*_*V*_ from pre- and trans-saccadic target localization with respect to the actually performed saccade. This revealed that visual prediction error is not nullified during learning. If the visuomotor system nullified visual prediction error, this would mean that after adaptation asymptote has been reached, the predicted visual error matches the actual visual error after saccade landing. Hence, subjects would predict the target to appear after the saccade (i.e. localize the pre-saccadic target after saccade landing in the dark) where the target actually appeared in saccade trials when a post-saccadic target is shown. This was obviously not the case. However, learning was well explained by nullification of postdictive motor error. This error relies on a postdictive update of the pre-saccadic target position based on post-saccadic visual input and computes the saccade error with respect to this position. Supporting this idea, in Heins et al. (2023) [93] we have recently shown that adaptive changes to oculomotor behavior can be induced by post-saccadic visual information alone, i.e. without pre-saccadic visual information.

#### Pre-saccadic calculations

The pre-saccadic target position *V*_1_^(*n*)^ on the visuospatial map is scaled by the visual gain *ω*_*v*_^(*n*)^:

$$\begin{array}{c} V_{1}{}^{\left(n\right)}=P_{1}\omega_{v}{}^{\left(n\right)} \end{array}$$ (1)

with the trial number *n* and physical target eccentricity *P*_1_ (Fig 1B-1). If *ω*_*v*_^(*n*)^ = 1, the target is accurately localized on the visuospatial map. If a postdictive motor error is assigned to an internal failure of visuospatial target representation, *ω*_*v*_^(*n*)^ is adapted to reduce the error for future movements (see Eq 14).

The inverse model (Fig 1B-2) transforms the pre-saccadic target position into a motor command with the motor gain *ω*_*m*_^(*n*)^:

$$\begin{array}{c} M{}^{\left(n\right)}=V_{1}{}^{\left(n\right)}\omega_{m}{}^{\left(n\right)}=P_{1}\omega_{v}{}^{\left(n\right)}\omega_{m}{}^{\left(n\right)} \end{array}$$ (2)

Thus, the inverse model transforms the movement goal from visuospatial to motor coordinates based on the assumed state of muscle dynamics. If *ω*_*m*_^(*n*)^ = 1, the saccade lands at the spatial position *V*_1_^(*n*)^. If a postdictive motor error is assigned to a change in muscle dynamics, *ω*_*m*_^(*n*)^ is adapted to reduce the error for future movements (see Eq 14). The quantification of changes to the visual gain *ω*_*v*_, on the one hand, and the motor gain *ω*_*m*_, on the other hand, dissociates the contribution of the two processes to behavioral output, i.e. saccade vector change.

The motor command *M*^(*n*)^ is copied into *CD*_*M*_^(*n*)^:

$$\begin{array}{c} CD_{M}{}^{\left(n\right)}=M{}^{\left(n\right)} \end{array}$$ (3)

and directed into the CD pathway.

Before saccade start, the forward dynamics model (Fig 1B-3) transforms the corollary discharge of the motor command *CD*_*M*_^(*n*)^ into the *CD*_*V*_^(*n*)^ signal, i.e. the computed displacement of visual space, to inform the visual system about the upcoming saccade:

$$\begin{array}{c} CD_{V}{}^{\left(n\right)}=CD_{M}{}^{\left(n\right)}\omega_{cd}{}^{\left(n\right)}=P_{1}\omega_{v}{}^{\left(n\right)}\omega_{m}{}^{\left(n\right)}\omega_{cd}{}^{\left(n\right)} \end{array}$$ (4)

If *ω*_*cd*_^(*n*)^ = 1, *CD*_*V*_^(*n*)^ matches the actual saccade vector. If a postdictive motor error is assigned to an internal failure of *CD*_*V*_ representation, *ω*_*cd*_^(*n*)^ is adapted to reduce the error for future movements (see Eq 14).

Based on *CD*_*V*_^(*n*)^, the forward outcome model (Fig 1B-4) shifts the coordinates of the visual pre-saccadic target position *V*_1_^(*n*)^ to predict the visual post-saccadic target position:

$$\begin{array}{c} {\hat{V}}_{2}{}^{\left(n\right)}=V_{1}{}^{\left(n\right)}-CD_{V}{}^{\left(n\right)}=P_{1}\omega_{v}{}^{\left(n\right)}\left(1-\omega_{m}{}^{\left(n\right)}\omega_{cd}{}^{\left(n\right)}\right) \end{array}$$ (5)

If *CD*_*V*_^(*n*)^ is correct, i.e. if it matches the actual saccade vector, pre- and post-saccadic visual scene are correctly integrated such that the post-saccadic target is predicted to appear (${\hat{V}}_{2}{}^{\left(n\right)}$) at the same spatiotopic position where the pre-saccadic target was perceived (*V*_1_). In the experiment, this is the case when the flash is localized at the same position in the pre- and post-saccadic localization trials.

#### Saccade kinematics

Saccadic motor learning has been shown to be differently transposed to saccade kinematics, i.e. to saccade peak velocity and saccade duration, depending on the error direction [42, 56]. Moreover, there is evidence that cerebellar patients are more impaired in learning from outward than from inward errors [42]. Thus, capturing the learning effect on the patients’ saccade kinematics may provide a deeper understanding of how the cerebellum operates the recalibration of motor commands. Hence, we expanded the model of Masselink et al. (2021) [38] with how the motor command is transposed to saccade peak velocity and saccade duration. Accordingly, the saccade is executed with the saccade peak velocity *κ*^(*n*)^ and the saccade duration λ^(*n*)^ that are specific for the motor command (Fig 1B-5, see Eqs 16, 18 and 20 for how the motor command is transposed to saccade peak velocity and saccade duration depending on the paradigm). This specificity relies on a linear relationship between a saccade’s amplitude, its peak velocity and its duration, known as the saccadic main sequence [51, 104, 105]. Hence, we specify the relationship between the motor command *M*^(*n*)^, the saccade peak velocity *κ*^(*n*)^ and the saccade duration λ^(*n*)^ by the following equation:

$$\begin{array}{c} M{}^{\left(n\right)}=\beta_{0}+\beta_{\kappa }\kappa {}^{\left(n\right)}+\beta_{\lambda }\lambda {}^{\left(n\right)} \end{array}$$ (6)

For the modeling, we derive *β*_0_, *β*_*κ*_ and *β*_λ_ from fitting the equation to the saccade vectors, saccade peak velocities and saccade durations of patients and healthy controls. Please note that while the saccadic main sequence is usually expressed by two functions describing the relationship of the saccade amplitude with saccade peak velocity and saccade duration separately [51, 103], we unite this relationship in one single function. This allows us to model oculomotor fatigue compensation, i.e. when a decrease of saccade peak velocity does not inevitably result in a decrease of the saccade amplitude but can be compensated by a longer saccade duration such that the saccade amplitude stays stable. Two separate functions expressing the relationship of the saccade amplitude, first, to peak velocity and, second, to duration would not allow to model oculomotor fatigue compensation. We used a linear approximation in sort of a 3D plane as described by Eq 6 that appeared well justified over the rather small range of saccade amplitudes that we measured (Figs 3 and 4).

#### Saccade execution

The execution of the motor command produces the physical saccade vector (Fig 1B-6):

$$\begin{array}{c} P_{M}{}^{\left(n\right)}=M{}^{\left(n\right)}+\epsilon_{m}{}^{\left(n\right)}=P_{1}\omega_{v}{}^{\left(n\right)}\omega_{m}{}^{\left(n\right)}+\epsilon_{m}{}^{\left(n\right)} \end{array}$$ (7)

with random motor noise *ϵ*_*m*_^(*n*)^.

In the inward and outward learning paradigms, the target is shifted during saccade execution. The peri-saccadic target displacement due to the target shift *P*_*s*_ and the motor execution noise *ϵ*_*m*_ is described by:

$$\begin{array}{ccc} P_{d}{}^{\left(n\right)} & = & P_{s}-\epsilon_{m}{}^{\left(n\right)} \end{array}$$ (8)

#### Post-saccadic calculations

After saccade offset, the post-saccadic target is localized on the visuospatial map (Fig 1B-7):

$$\begin{array}{c} V_{2}{}^{\left(n\right)}=P_{1}+P_{d}{}^{\left(n\right)}+\epsilon_{m}{}^{\left(n\right)}-P_{M}{}^{\left(n\right)}=P_{1}\left(1-\omega_{v}{}^{\left(n\right)}\omega_{m}{}^{\left(n\right)}\right)+P_{d}{}^{\left(n\right)} \end{array}$$ (9)

Please note that the visual gain *ω*_*v*_^(*n*)^ is not applied to *V*_2_^(*n*)^ because visuospatial changes during saccadic motor learning underlie an adaptation field [24, 25]. Hence, a change in the visual gain *ω*_*v*_^(*n*)^ is a local effect around the pre-saccadic target position and should not be applied to the representation of the post-saccadic target after saccade landing.

The backward outcome model integrates the visual post-saccadic target position *V*_2_^(*n*)^ with *CD*_*V*_^(*n*)^ to from a postdictive update of the target position in a pre-saccadic reference frame (Fig 1B-8):

$$\begin{array}{c} {\hat{V}}_{1}{}^{\left(n\right)}=V_{2}{}^{\left(n\right)}+CD_{V}{}^{\left(n\right)}=P_{1}\left(1+\omega_{v}{}^{\left(n\right)}\omega_{m}{}^{\left(n\right)}\left(\omega_{cd}{}^{\left(n\right)}-1\right)\right)+P_{d}{}^{\left(n\right)} \end{array}$$ (10)

We refer to this process as the backward outcome model as it performs a postdictive transformation from post- to pre-saccadic space, analogously to the forward outcome model that performs a predictive transformation, i.e. from pre- to post-saccadic space.

#### Learning of visuomotor gains and its transposition to saccade kinematics

In the inward and outward learning paradigms, the visuomotor system experiences a significant motor error due to the peri-saccadic target step. This error, termed the postdictive motor error *E*_*post*_^(*n*)^, is calculated as the error of the motor command with respect to the postdicted pre-saccadic target position (Fig 1A-9):

$$\begin{array}{c} E_{post}{}^{\left(n\right)}={\hat{V}}_{1}{}^{\left(n\right)}-M{}^{\left(n\right)}=P_{1}\left(1+\omega_{v}{}^{\left(n\right)}\omega_{m}{}^{\left(n\right)}\left(\omega_{cd}{}^{\left(n\right)}-2\right)\right)+P_{d}{}^{\left(n\right)} \end{array}$$ (11)

To reduce *E*_*post*_^(*n*)^ on the next trial, the visuomotor system adapts its gains $\omega^{\left(n\right)}=\left(\begin{array}{c} \omega_{v}{}^{\left(n\right)}\\ \omega_{m}{}^{\left(n\right)}\\ \omega_{cd}{}^{\left(n\right)} \end{array}\right)$ following an internal estimate of the *E*_*post*_^(*n*)^ gradient [15, 94, 106]. We express this principle by the delta rule [107, 108]:

$$\begin{array}{ccc} \omega {}^{\left(n+1\right)} & = & \omega {}^{\left(n\right)}-\alpha \frac{\partial |E_{post}{}^{\left(n\right)}{|}^{2}}{\partial \omega {}^{\left(n\right)}} \end{array}$$ (12)

$$\begin{array}{ccc} & = & \omega {}^{\left(n\right)}-2\alpha E_{post}{}^{\left(n\right)}\frac{\partial E_{post}{}^{\left(n\right)}}{\partial \omega {}^{\left(n\right)}} \end{array}$$ (13)

$$\begin{array}{ccc} & = & \omega {}^{\left(n\right)}-2\alpha E_{post}{}^{\left(n\right)}(\begin{array}{c} P_{1}\omega_{m}{}^{\left(n\right)}\left(\omega_{cd}{}^{\left(n\right)}-2\right)\\ P_{1}\omega_{v}{}^{\left(n\right)}\left(\omega_{cd}{}^{\left(n\right)}-2\right)\\ P_{1}\omega_{v}{}^{\left(n\right)}\omega_{m}{}^{\left(n\right)} \end{array}) \end{array}$$ (14)

The learning rates $\alpha =(\begin{array}{ccc} \alpha_{v} & 0 & 0\\ 0 & \alpha_{m} & 0\\ 0 & 0 & \alpha_{cd} \end{array})\geq$ 0 specify the speed of learning.

The changes in the visual gain *ω*_*v*_ and in the motor gain *ω*_*m*_ result in a changed motor command *M*^(*n*+1)^ on the next trial. The transposition of the changed motor command to saccade peak velocity and saccade duration needs to take into account whether the motor command was decreased or increased, i.e. whether changes occurred during inward or during outward learning [42, 56]. Saccade shortening during inward learning is usually characterized by a decline of peak velocity while saccade duration stays constant. Accordingly, during inward learning in our model (*P*_*s*_ < 0), saccade duration λ stays constant while saccade peak velocity *κ* is downregulated on the next trial according to the changed motor command (Fig 1B-6):

$$\begin{array}{ccc} \lambda {}^{\left(n+1\right)} & = & \lambda {}^{\left(n\right)} \end{array}$$ (15)

$$\begin{array}{ccc} \kappa {}^{\left(n+1\right)} & = & \frac{M{}^{\left(n+1\right)}-\beta_{0}-\beta_{\lambda }\lambda {}^{\left(n+1\right)}}{\beta_{\kappa }} \end{array}$$ (16)

By contrast, saccade lengthening during outward learning is usually characterized by an increase of saccade duration while saccade peak velocity stays constant [42, 56]. Accordingly, during outward learning in our model (*P*_*s*_ > 0), saccade peak velocity *κ* stays constant while saccade duration λ is upregulated on the next trial according to the changed motor command (Fig 1B-6):

$$\begin{array}{ccc} \kappa {}^{\left(n+1\right)} & = & \kappa {}^{\left(n\right)} \end{array}$$ (17)

$$\begin{array}{ccc} \lambda {}^{\left(n+1\right)} & = & \frac{M{}^{\left(n+1\right)}-\beta_{0}-\beta_{\kappa }\kappa {}^{\left(n+1\right)}}{\beta_{\lambda }} \end{array}$$ (18)

The visuomotor system reaches a steady state, i.e. learning comes to an end, if the saccade lands at the postdicted pre-saccadic target position such that *E*_*post*_^(*n*)^ = 0.

#### Oculomotor fatigue and its compensation by saccade duration

For a deeper understanding of which calibration processes are perturbed due to cerebellar disease, we also modeled the experimental condition without any target step, i.e. without learning. Although target localizations and saccade vectors should stay roughly constant in healthy subjects, the repetitive execution of saccades to the same target position is usually accompanied by a decline in saccade peak velocity, a sign of oculomotor fatigue. However, this compensating mechanism has been shown to be impaired in patients with vermal cerebellar lesion [42]. Accordingly, in the no step condition of our model (*P*_*s*_ = 0), saccade peak velocity *κ* declines while saccade duration λ compensates by a certain percentage on the next trial (Fig 1B-6):

$$\begin{array}{ccc} \kappa {}^{\left(n+1\right)} & = & \kappa {}^{\left(n\right)}-\gamma_{\kappa }\;(\kappa {}^{\left(n\right)}-\kappa_{end}) \end{array}$$ (19)

$$\begin{array}{ccc} \lambda {}^{\left(n+1\right)} & = & \lambda {}^{\left(n\right)}-\gamma_{\lambda }\;(\lambda {}^{\left(n\right)}-\frac{(M{}^{\left(n\right)}-\beta_{0}-\beta_{\kappa }\kappa {}^{\left(n+1\right)}}{\beta_{\lambda }}) \end{array}$$ (20)

Here, *γ*_*κ*_ ≥ 0 describes the decay rate of peak velocity, *κ*_*end*_ limits peak velocity loss and *γ*_λ_ describes the percentage by which saccade duration compensates for the peak velocity loss (0 ≤ *γ*_λ_ ≤ 1). According to the changes of peak velocity *κ* and duration λ in the no step condition (*P*_*s*_ = 0), the motor gain *ω*_*m*_^(*n*+1)^ of the next trial is:

$$\begin{array}{c} \omega_{m}{}^{\left(n+1\right)}=\frac{\beta_{0}+\beta_{\kappa }\kappa {}^{\left(n+1\right)}+\beta_{\lambda }\lambda {}^{\left(n+1\right)}}{P_{1}\omega_{v}{}^{\left(n+1\right)}} \end{array}$$ (21)

Hence, during expected oculomotor fatigue in the no step condition, we model changes in saccade peak velocity and saccade duration that are reflected in the motor gain *ω*_*m*_ while *ω*_*v*_ and *ω*_*cd*_ are assumed to stay stable.

### Analysis

#### Data processing

Data analysis was performed in Matlab R2017a (Mathworks, Natick, MA). Saccades were detected offline with a self-made routine that searched for a saccade after target onset with an onset and offset velocity threshold of 80 deg/s. Saccade traces as well as onset and offset detection were visualized trial by trial in order to correct for detection errors. Based on the verified saccade onset and offset, saccade latency, peak velocity and duration were extracted. The horizontal eye position signal was smoothed and the saccade start position was determined based on the position 50 ms prior to saccade onset detection. The saccade end position was extracted based on the position 50 ms after saccade offset detection to prevent post-saccadic oscillation effects.

Saccades with a latency of less than 100 ms, an amplitude below 10° or above 30° as well as localizations with more than ±7° localization error were excluded from the analysis. Moreover, as our modeling approach explicitly relies on the localization judgement of target eccentricity, we chose only the pre- and post-saccadic localization trials in which the flash position matched the saccade target position, i.e. 20° eccentricity. Thus, we ensured that our modeling approach quantifies perceptual changes exactly at the spatial position of the target and is not disturbed by the process of learning transfer to nearby locations, known as the saccadic adaptation field [12, 24, 25, 52, 60, 69, 109, 110].

For each condition of each subject, we calculated the mean saccade vector, mean saccade peak velocity and mean saccade duration of the 20 pre-exposure saccade trials and the last 20 exposure saccade trials, respectively. The removal of invalid trials resulted in 39.3 ± 2.7 used saccade trials in control subjects and 33.2 ± 3.8 used saccade trials in patients (summed across the pre-exposure and late exposure phase averaged across the three conditions; difference between control subjects and patients *t*_14_ = 1.97, *p* = .069).

For the pre- and the post-saccadic flash localizations we extracted the mean of the pre-exposure and the post-exposure phase. These were eight trials as we did only include those in which the flash matched the distance of the target, i.e. 20° eccentricity. The removal of invalid trials resulted in 7.8 ± 0.5 used pre-saccadic localization trials in control subjects and 7.5 ± 0.8 used pre-saccadic localization trials in patients (difference between control subjects and patients *W* = 75.5, *p* = .446). From the post-saccadic localization trials, 7.5 ± 0.2 trials were used in the control group and 7.5 ± 0.5 trials were used in the patients group (difference between control subjects and patients *W* = 64.5, *p* = .668).

In addition, we quantified a cross-condition baseline state for each subject, averaging the mean saccade vectors, saccade peak velocities, saccade durations and pre- and post-saccadic localizations across the three conditions. Outliers with more than three standard deviations from the mean were excluded. We averaged the saccade vectors, pre- and post-saccadic localizations at group level, i.e. separately for patients and control subjects and condition. Moreover, we calculated the mean trial-by-trial saccade vectors, saccade peak velocities and saccade durations of the 120 exposure trials, separately for patients and control subjects. After invalid trials were removed, 117.3 ± 11.1 saccade trials could be used in the control group and 96.9 ± 11.1 saccade trials could be used in the patient group (difference between control subjects and patients, Wilcoxon rank sum test, *W* = 86.0, *p* = .065).

#### Model-based analysis

We calculated the state of the *CD*_*V*_ signal, the visuomotor gains ***ω*** and the *E*_*post*_ error of each subject’s pre-exposure and post-exposure phase. Based on the pre-saccadic target localization (*V*_1_), the saccade vector (*M*) and the post-saccadic target localization with respect to the saccade landing position (${\hat{V}}_{2}$), we derive:

$$\begin{array}{ccc} CD_{V} & = & V_{1}-{\hat{V}}_{2} \end{array}$$ (22)

$$\begin{array}{ccc} \omega_{v} & = & \frac{V_{1}}{P_{1}} \end{array}$$ (23)

$$\begin{array}{ccc} \omega_{m} & = & \frac{M}{V_{1}} \end{array}$$ (24)

$$\begin{array}{ccc} \omega_{cd} & = & \frac{CD_{V}}{CD_{M}} \end{array}$$ (25)

$$\begin{array}{ccc} E_{post} & = & P_{1}\left(1+\omega_{v}\omega_{m}\left(\omega_{cd}-2\right)\right)+P_{d} \end{array}$$ (26)

with *P*_1_ = 20° and *P*_*d*_ = 0° for the pre-exposure phase and the post-exposure phase of the no step condition, *P*_*d*_ = -6° for the post-exposure phase of the inward condition and *P*_*d*_ = 6° for the post-exposure phase of the outward condition. Additionally, we derived the change of each variable from the pre- to the post-exposure phase.

Before fitting the model to the trial-by-trial data, we performed a regression of the saccade vectors on saccade peak velocities and saccade durations of the exposure phase, separately for each group (controls, patients) and each condition (inward, no step, outward). See Table 2 for the fitted regression weights.

Starting from the pre-exposure mean of pre-saccadic localization (*V*_1_^(1)^), post-saccadic localization (with respect to the saccade landing point, ${\hat{V}}_{2}{}^{\left(1\right)}$), saccade vector (*M*^(1)^), saccade peak velocity (*κ*^(1)^) and saccade duration (λ^(1)^) taken as values of trial *n* = 1, we fitted the model to the exposure phase, separately for each group (control subjects, patients) and each condition (inward, no step, outward). For the inward and the outward condition, we fitted the learning rates of the visuomotor gains (***ϕ*_*fit*_** = ***α***), and for the no step condition, we fitted the peak velocity decay rate and the duration compensation rate (***ϕ*_*fit*_** = (*γ*_*κ*_, *γ*_λ_)). The fitting procedure minimized the weighted sum of squared errors (SSE):

$$\begin{array}{ccc} \phi_{fit} & = & argmin(\sum_{1,120}{\left(\eta_{V_{1}}{\left(V_{1}{}^{\left(n\right)}-V_{1,predicted}{}^{\left(n\right)}\right)}^{2}+\eta_{{\hat{V}}_{2}}({\hat{V}}_{2}{}^{\left(n\right)}-{\hat{V}}_{2,predicted}{}^{\left(n\right)}\right)}^{2})\\ & & +\sum_{1}^{120}{\left(\eta_{M}(M{}^{\left(n\right)}-M_{predicted}{}^{\left(n\right)}\right)}^{2}+\eta_{\kappa }(\kappa {}^{\left(n\right)}-\kappa_{predicted}{}^{\left(n\right)}{)}^{2}+\eta_{\lambda }(\lambda {}^{\left(n\right)}-\lambda_{predicted}{}^{\left(n\right)}{)}^{2})) \end{array}$$

with the weights $\eta_{V_{1}}$ = $\eta_{{\hat{V}}_{2}}=90.090$, *η*_*M*_ = 0.015, *η*_*κ*_ = 0.012 and *η*_λ_ = 0.003 to account for the unequal number of data points the signals were fitted to (e.g. only two data points for *V*_1_ and ${\hat{V}}_{2}$ but 120 data points for *M*, *κ* and λ) and for the unequal units (e.g. a saccade vector error of 3° should be weighted more than a peak velocity error of 3$\frac{{}^{{}^{\circ}}}{s}$). In the inward and in the outward condition patients showed a small *V*_1_ change in the opposite direction of learning. This effect was small, not significant and, thus, rather of random nature (see Fig 5B). In these two cases, we fitted a stable *V*_1_ as the mean of the pre-saccadic target localizations between the pre- and the post-exposure phase. For all model fits, we set *ϵ*_*m*_^(*n*)^ = 0.

For goodness of fit, residual standard errors *RSE* were calculated, collectively for *V*_1_, *M* and ${\hat{V}}_{2}$ (in °) and separately for *κ* (in $\frac{{}^{{}^{\circ}}}{s}$) and λ (in ms):

$$\begin{array}{c} RSE=\sqrt{\frac{SSE}{q-1}} \end{array}$$ (27)

with *q* as the number of data points used for the respective *RSE* calculation.

#### Simulation of long-term visuomotor behavior after disease onset

We simulated how the visuomotor system would behave on a long timescale after the onset of cerebellar disease, i.e. when a fatigue-induced loss of saccade peak velocity *κ* can be only partially compensated by upregulation of saccade duration λ while visuomotor learning is progressively reduced. Simulations were performed for a target of *P*_1_ = 10° eccentricity, ***ω***^(1)^ = (0.958; 1.023; 0.980) (which are the baseline gains measured in healthy subjects of Masselink et al. (2021) [38]), *κ*^(1)^ = 300$\frac{{}^{{}^{\circ}}}{s}$, λ^(1)^ = 43.9 ms, *γ*_*κ*_^(1)^ = 0.003, *γ*_λ_^(1)^ = 0.964 (derived from the control subjects’ model fit to the no step condition), *κ*_*end*_ = 200$\frac{{}^{{}^{\circ}}}{s}$, *α*_*v*_^(1)^ = 2.35*10^−6^, *α*_*m*_^(1)^ = 5.53*10^−6^, *α*_*cd*_^(1)^ = 5.31*10^−13^ (derived from control subjects’ model fit to the outward condition), *β*_0_ = -11.67, *β*_*κ*_ = 0.03, *β*_*κ*_ = 0.27 (derived from the saccade vectors, saccade peak velocities and saccade durations of control subjects across all three conditions to capture a minimum range of saccade vectors). The progressive impairment of oculomotor control parameters was reflected by a successive change of the peak velocity decay rate *γ*_*κ*_, the duration compensation rate *γ*_λ_ and the learning rates ***α*** towards patients’ values:

$$\begin{array}{ccc} \gamma_{\kappa }{}^{\left(n+1\right)} & = & \gamma_{\kappa }{}^{\left(n\right)}-\nu_{\kappa }\;(\gamma_{\kappa }{}^{\left(n\right)}-\gamma_{\kappa ,end}) \end{array}$$ (28)

$$\begin{array}{ccc} \gamma_{\lambda }{}^{\left(n+1\right)} & = & \gamma_{\lambda }{}^{\left(n\right)}-\nu_{\lambda }\;(\gamma_{\lambda }{}^{\left(n\right)}-\gamma_{\lambda ,end}) \end{array}$$ (29)

$$\begin{array}{ccc} \alpha {}^{\left(n+1\right)} & = & \alpha^{\left(n\right)}-\nu_{\alpha }\;(\alpha {}^{\left(n\right)}-\alpha_{end}) \end{array}$$ (30)

The progressive change proceeded until (1) the oculomotor fatigue parameters matched those obtained from the patients’ model fit to the no step condition (*γ*_*κ*,*end*_ and *γ*_λ,*end*_), and (2) until the learning rates matched those obtained from the patients’ model fit to the outward condition (***α***_*end*_). The progression rates *ν*_*κ*_, *ν*_λ_ and *ν*_*α*_ determine how fast the respective parameters change from healthy controls’ to patients’ values. We examined which range of progression rates can mirror the course of the disease such that the oculomotor system converges at the patients’ baseline gains ***ω***, hence, leading to an outward localization of visual targets. Thereby, we tested which parameter combinations lead to a residual standard error *RSE*_*ω*_ ≤ 0.05 at the end of the simulation with respect to the patients’ baseline gains ***ω***. This corresponds to a mean gain deviation below 4% from the patients’ baseline gains at the end of the simulation. For each simulation, we computed the progression of the disease across 23,328 Mio saccades. With 3 saccades per second and 12 awake hours per day this roughly corresponds to the number of saccades that are performed across 6 months. Though, please note that 6 months in our simulation would correspond to a multiple of 6 months in real life as in real life we perform saccades of different amplitudes and different directions. Hence, our simulation is a strong simplification of the changes in oculomotor control during cerebellar disease progression and rather examines relative timescale differences between the changes in peak velocity decay, duration compensation and learning rates.

#### Statistical analysis

To compare the data of one group against zero, we used two-sided one-sample t-tests or a Wilcoxon signed rank test in case normality distribution was violated. To compare data between groups, i.e. between patients and control subjects, two-sided two-sample t-tests, or, alternatively, Wilcoxon rank sum tests in case normality distribution was violated, were calculated. All tests were conducted with a significance level of 0.05.
